# Removal of trace gases can both increase and decrease cloud droplet formation

**DOI:** 10.1126/sciadv.adx0960

**Published:** 2026-01-14

**Authors:** Elavarasi Ravichandran, Sanghee Han, Abigail S. Williams, Veronica Berta, Jeremy L. Dedrick, Christian Pelayo, Nattamon Maneenoi, Lynn M. Russell, Michael Wheeler, Jeremy Wentzell, John Liggio, Markus D. Petters

**Affiliations:** ^1^ Department of Chemical and Environmental Engineering, University of California, Riverside, Riverside, CA 92507, USA.; ^2^ Center for Environmental Research and Technology (CE-CERT), University of California, Riverside, Riverside, CA 92507, USA.; ^3^ Scripps Institution of Oceanography, University of California, San Diego, La Jolla, CA 92037, USA.; ^4^ Air Quality Research Division, Environment and Climate Change Canada, Toronto M3H 5T4, Canada.

## Abstract

Aerosols consist of liquid or solid particles dispersed in a gas. Aerosol measurements generally rely on drying the particles before quantifying their physicochemical properties. This drying can potentially remove semivolatile compounds from the particles. Here, we show size-resolved cloud condensation nuclei (CCN) measurements quantifying the hygroscopicity parameter in the presence and absence of a denuder. The denuder efficiently removed alkanes and weakly functionalized acids, aldehydes, and alcohols with fewer than 10 carbon atoms from the gas phase. Denuding organic compounds perturbed the CCN-derived hygroscopicity parameter by up to 50%. Denuding either rendered the particles more or less CCN active, and the direction of the effect depended on sample relative humidity and trace gas concentration. The effect was weakest in early spring and strongest in late spring and summer. The measurements demonstrate an unexpectedly strong coupling between the particle and gas phase, influencing CCN activity through either volatilization or surface adsorption, or both.

## Teaser

Rapid repartitioning of semivolatile compounds can strongly alter the potential of an aerosol to nucleate cloud droplets.

## INTRODUCTION

Cloud droplet number concentrations in shallow low-lying liquid-water clouds are strongly susceptible to the addition of submicrometer-sized cloud condensation nuclei (CCN) (*1*, *2*). In these clouds, the addition of CCN generally results in an increase in droplet number concentration, cloud albedo, cloud lifetime, and cloud fraction, and a decrease in precipitation efficiency (*1*–*3*). Particles from human activity, including those from continental sources and ship emissions, have modified cloud albedo to an extent substantial enough to offset some of the climate warming caused by CO_2_, although the exact amount remains highly uncertain (*4*). The purposeful addition of CCN to regions with low-lying liquid clouds is widely discussed as a potential avenue for geoengineering through marine cloud brightening (*5*, *6*). Köhler theory links the particle size and chemical composition to the water supersaturation (*S*) required for a particle to serve as CCN at equilibrium (*7*). When Köhler theory is combined with size distribution and size-resolved chemical composition measurements, it is possible to calculate the CCN activation spectrum, defined as the cumulative number concentration of CCN as a function of water supersaturation (*8*). The CCN activation spectrum can also be measured directly with CCN instruments (*9*–*11*). Adiabatic cloud parcel models further link the CCN spectrum and updraft velocity to obtain cloud droplet number concentrations (*12*–*15*). A fundamental scientific understanding of the effect of aerosol on clouds requires—at minimum—closure between aerosol size, aerosol composition, and calculated CCN spectra with observed CCN spectra.

The general approach for characterizing the aerosol-to-CCN link has been to dry the aerosol stream before measuring aerosol size and composition due to the difficulty of quantifying particle-bound water mass when sizing at elevated relative humidity (RH) (*16*). However, semivolatile compounds associated with the aqueous phase, dissolved gases, and surface adsorbed gases may be lost by particle drying (*6*, *17*, *18*), resulting in a change in the critical supersaturation required to activate those particles into cloud droplets. The critical supersaturation is controlled by the amount of dissolved compounds inside the droplet and the surface tension of the droplet. Common dissolved species present in the aqueous aerosol include sulfates and organic compounds (*19*). Volatile water-soluble inorganic gases such as nitric acid can partition into the growing droplet and in turn add to the dissolved species. Furthermore, the addition of nitric acid may also lead to dissolution of sparingly soluble compounds that were already present in the particle, thus further adding to the amount dissolved in the droplet (*20*, *21*). Organic trace gases such as methylglyoxal and acetaldehyde can adsorb to the water/air interface and reduce the surface tension of the droplet (*22*). Condensation of semivolatile organic vapors during adiabatic ascent, followed by dissolution into the droplet may also add to the dissolved solutes (*17*, *23*, *24*). Each of these effects is expected to lower the activation supersaturation relative to an aerosol in the absence of inorganic or organic trace gases. Although there is some evidence from laboratory experiments indicating that trace gases can affect the activation supersaturation (*22*, *24*, *25*), this effect has not been investigated in field studies.

Here, we test the hypothesis that removing semivolatile compounds from the particles by removing gas-phase compounds followed by reequilibration of the aerosol will alter the supersaturation required for the activation of ambient particles. To this end, CCN measurements were conducted from February through August 2023 near the peak of Mt. Soledad (32.8398°N, 117.2523°W), which is located in San Diego, CA, USA, a few kilometers inland from the Pacific coastline. The site is influenced by offshore marine sources as well as pollution sources transported from the Los Angeles metropolitan area. Typical transport times are 0.5 to 1.5 days, and transport from Los Angeles to the site often includes substantial processing by persistent stratocumulus clouds over the Pacific Ocean in this coastal region (*12*, *26*, *27*), which are sampled as part of this study. The mobility size distribution and size-resolved CCN-derived hygroscopicity (κ) (*7*) were measured by interfacing a scanning mobility particle spectrometer with a condensation particle counter and a continuous-flow CCN counter (CCNc) sampling between 0.2 and 1% supersaturation. During alternating size distribution scans, particles exiting the differential mobility analyzer (DMA) column were passed through a denuder that removed alkanes and weakly functionalized acids, aldehydes, and alcohols with fewer than 10 carbon atoms with more than 99% efficiency from the gas phase (fig. S1).

## RESULTS

The activation supersaturation of a particle is a function of the particle’s dry diameter and chemical composition. The hygroscopicity parameter, κ, is derived from the size-resolved CCN measurements (Material and Methods), and the κ value expresses the intrinsic capacity of a particle to activate into a droplet. Larger values correspond to lower activation supersaturation for the same amount of dissolved compounds. Alternatively, κ can be estimated from hygroscopic water content measurements from a humidified tandem DMA (HTDMA) at RH = 85% (Material and Methods). These two measurements are referred to as subsaturated κ and CCN-derived κ, respectively. For ideal solutions, subsaturated κ and CCN-derived κ should be identical. For this dataset, the HTDMA-derived and CCN-derived κ covaried. However, the CCN-derived κ exceeded the subsaturated κ by up to ~50% (fig. S9). Such discrepancies between HTDMA-derived and CCN-derived κ are often observed for colocated measurements around the world, and the discrepancy here is well within the range of several prior field studies (*28*–*33*).

### The effect of denuding on CCN-derived κ

**Fig. 1.**
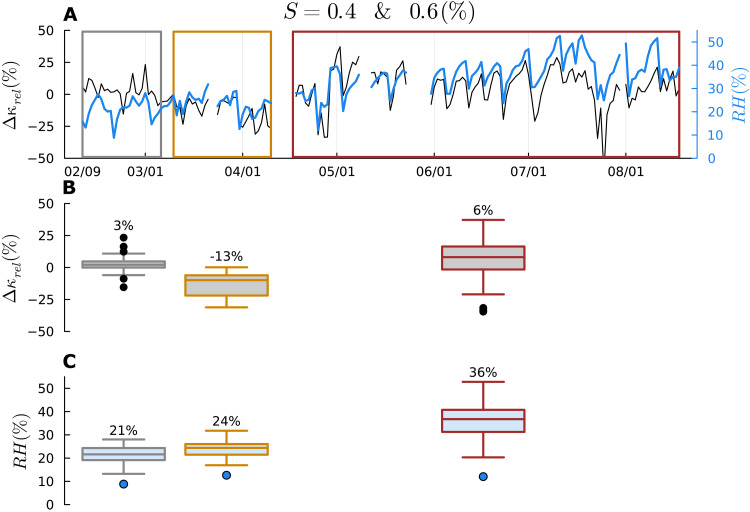
Correlation between Δκ_rel_ and inlet RH*.* (**A**) Temporal variations in daily average Δκ_rel_ (black) and sample flow RH entering the CCN instrument (blue) from February to August in 2023 for supersaturations 0.4 and 0.6%. Date ticks are formatted as month/day. Three periods are highlighted in gray, orange, and brown. (**B** and **C**) Box-and-whisker plots indicating the minimum, first quartile, median, third quartile, and maximum within each period. The interquartile range (IQR) is used to identify outliers. Data points that are more than 1.5 times the IQR are considered outliers. Minimum and maximum are determined with the outliers removed. Percentage values above the box plot correspond to the median values for each period for Δκ_rel_ and RH.

Passage through the denuder either decreased or increased the CCN-derived κ (Fig. 1), with the relative difference between the undenuded and denuded hygroscopicity (hereafter Δκ_rel_) varying between approximately −25% and +25%. The main hypothesis explaining the observed Δκ_rel_ is that the denuder scrubs the gas phase and either adsorbed or absorbed compounds partially or fully reequilibrate within the ~20-s transit time between the exit of the denuder and exposure to the maximum supersaturation inside the CCN.

### Period-based analysis of Δκ_rel_ variability

The observations were subdivided into three time periods. During time period 1 (gray), the median Δκ_rel_ ≈ 0 and the variability in Δκ_rel_ was smaller than the other periods. During time period 2 (orange), denuding increased the κ of the aerosol, with the median Δκ_rel_ ≈ −13%. The time series shows a noisy but systematically decreasing trend with time. During time period 3 (brown), the median Δκ_rel_ ≈ 6%. Notably, Δκ_rel_ is substantially more variable compared to periods 1 and 2. Figure 1 indicates clear covariation between Δκ_rel_ and the RH measured at the inlet of the size-resolved CCN instrument during periods 2 and 3. For example, the Pearson correlation coefficient between Δκ_rel_ and inlet RH is *R*^2^ = 0.55 for 6-hour averaged data from 31 May to 06 July 2023 for *S* = 0.4 and 0.6% (fig. S12). At inlet RH values exceeding 40%, denuding decreased κ, resulting in Δκ_rel_ > 25%.

Sample flow RH at the inlet increased gradually because of reduction in the efficiency of the silica gel dryer that removed water vapor from the inlet flow. It sharply decreased when the silica gel was changed, which occurred when RH reached 40 to 50% at approximately weekly intervals in spring and summer. Note that the median RH was similar during time period 1 and period 2. Nonetheless, the median Δκ_rel_ decreased during period 2. This suggests that the influence of the denuder on Δκ_rel_ depends not only on RH but also on the composition of either the aerosol or the gas phase or both. The data in Fig. 1 and associated discussion are limited to *S* = 0.4 and 0.6% (activation diameter ~ 63 ± 10 nm, *n* = 9621). The correlation between Δκ_rel_ and RH is poorest for *S* = 0.2% (*R*^2^ = 0.15), strongest for *S* = 0.4 and 0.6% (*R*^2^ = 0.54), and weaker for *S* = 0.8 and 1% (*R*^2^ = 0.3). The *S* = 0.2% data were systematically different from the remaining data, and, therefore, we focus on *S* = 0.4 to 1% for the multivariate statistical analysis in the next section. Similar trends as in Fig. 1 are observed for *S* = 0.2% and *S* = 0.8 and 1.0% (figs. S13 and S14).

### Controlling factors for Δκ_rel_

Size selection at elevated RH implies that particles are hydrated in the DMA because the DMA sheath flow was operated in recirculating mode thus approximately tracking inlet RH. The added water content will bias the observed total κ low since the total solute content is reduced (*16*). Assuming that RH = 50% and a true κ = 0.3, the calculated biased κ is 0.24, −21.6% lower than the true value. However, Δκ_rel_ represents the differences between denuded and undenuded after passage through the DMA. Whether or not the particles are hydrated during size selection should not, to first approximation, alter the solute content and the expected Δκ_rel_ = 0. Thus, deviation from this null hypothesis indicates that the influence of the gas phase on CCN activity may depend on the history and hydration state of the particles.

**Fig. 2.**
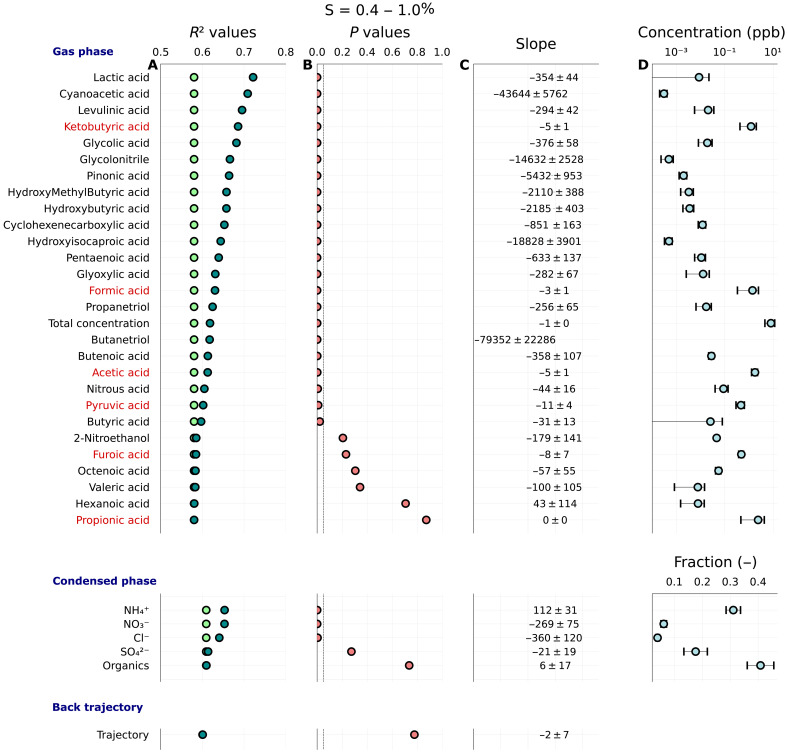
Change *R*^2^ in the regression result due to inclusion of a second explanatory variable. (**A**) Light green points correspond to the *R*^2^ regressing Δκ_rel_ against RH only. Green points correspond to the increased *R*^2^ from regressing against RH and the variable named on the left. (**B**) The *P* values give the probability that the increase in *R*^2^ is due to random chance, calculated using the *F* test. (**C**) The slope corresponds to the mean ± SE of the second regressed variable. (**D**) The absolute value mean ± SD of the second regressed variable. The dark red text highlights species that dominated gas-phase composition measured by the CIMS. Data used in the regression analysis are 6-hour average values collected between 31 May and 06 July 2023. Note that the *F* test model intercomparison requires identical datasets. Therefore, the intersection of missing values between the kappa and AMS and κ and CIMS datasets yields a slightly different *R*^2^ for regression of Δκ_rel_ with RH only.

Since the effect of denuding strongly depends on inlet RH and since the variation of inlet RH was so gradual and not explicitly controlled, tracking Δκ_rel_ at constant inlet RH versus time is challenging. To test for controlling factors that influence Δκ_rel_ other than RH, we contrasted regression models predicting Δκ_rel_ from only RH and Δκ_rel_ from RH and a second variable including gas-phase concentrations of the species measured by the chemical ionization mass spectrometer (CIMS, available for May and June only, dominant species were formic, acetic, propionic, keto butyric, pyruvic, and furoic acid), air mass origin through categorical back trajectory clusters, and the mass fractions of the five species measured by the aerosol mass spectrometer (nitrate, ammonium, sulfate, organics, and chloride). Regression assumed a functional form Δκ_rel_ = 1 *+* β_1_ × RH + β_2_ × *x*, where β_i_ are the slope parameters and *x* is the second regressed variable. This resulted in 34 evaluated models for the period when both gas phase and condensed phase chemical composition were available. Including a second variable increased the variance explained from 61%, considering RH only, up to ~65% considering RH and NH_4_^+^ fraction from the accelerator mass spectrometry (AMS). Considering RH and lactic acid from the CIMS increased the variance explained to 72%. The *F* test was performed to assess whether the increase in explanatory power by including a second parameter was statistically significant (*34*). Figure 2 summarizes the change in *R*^2^ values when including a second component, the *P* values, the slopes of the second regression coefficient, and the abundance of the material. Of the bulk composition variables tested, NH_4_^+^, NO_3_^−^, and Cl^−^ fraction were considered statistically significant at the 5% confidence level. Of the trace gas composition variables tested, 22 were statistically significant at the 5% confidence level. Among these were mostly organic acids. There is some degree of cross-correlation between the gas-phase compounds, thus leaving the possibility that a combination of one or more compounds is responsible for the effect of trace gases on Δκ_rel_. Three compounds are highlighted. Lactic acid had the largest increase in *R*^2^, cyanoacetic acid the largest negative slope, and keto-butyric acid representing one of the most abundant organic acids. Note that all of the identified statistically significant trace gases have a negative slope, indicating that an increase in trace gas concentration corresponds to a decrease in Δκ_rel_. This was further confirmed through ordinary regression of Δκ_rel_ versus trace gas concentration (fig. S15). The magnitude of the trace gas effect on Δκ_rel_, evaluated by multiplication of the regression slopes and mean trace gas concentration, varies between −3% for lactic acid and −13% for cyanoacetic acid. Assuming that cyanoacetic acid is solely responsible for the effect, Δκ_rel_ would change from 0 to −13% when changing cyanoacetic acid concentration from zero to the observed mean value, which is the same as the second period in Fig. 1. This is equivalent to the statement that removing trace gases by denuding increases κ. More directly, an increase in trace gas concentration depresses CCN activity.

Prior laboratory studies have identified nitric acid, methyl-glyoxal, acetaldehyde, and secondary organic aerosol oxidation products as gas-phase species (*22**,* *24**,* *35*), and amine secondary organic aerosol and ammonium nitrate (*35*, *36*) as semivolatile condensed phase species that may influence Δκ_rel_. The underlying mechanism for a species influence on Δκ_rel_ may be different for each species and is expected to be mediated by the presence of aerosol water (or ambient RH) since the presence of water may alter the bulk phase state and interfacial properties. Various mechanistic hypotheses explaining the observations are discussed next.

## DISCUSSION

The overall range in Δκ_rel_ from −25 to +25% induced by an ~20-s transit through a charcoal diffusion denuder tube is remarkable when considered in the context of other processes that are thought to modify κ under atmospheric conditions. For context, a 30% decrease in κ would correspond to a 10% decrease in particle diameter due to evaporation of semivolatile compounds inside the denuder, the kinetics of which is further discussed below. Furthermore, a 50% increase in κ for organic aerosol corresponds to an equivalent change in oxygen-to-carbon ratio of ~0.4 (*37*). While such changes can be simulated by laboratory conditions using oxidation flow reactors (*38*), the exposure corresponds to an atmospheric timescale of several weeks, and certainly such chemical changes do not occur during passage through the denuder. Heterogeneous chemical aging by OH oxidation yields much smaller changes in κ (*39*–*41*), except in rare circumstances where oxidation changes the solubility of organic compounds from effectively water insoluble to water soluble by addition of one or two functional groups (*42*, *43*). Thus the observation of a net 50% change in κ is large relative to other known chemical processes.

Evaporation can explain Δκ_rel_ > 0, corresponding to a decrease in κ after passage through the denuder. A 30% decrease in κ due to evaporation corresponds to an ~30% reduction in particle volume or 10% reduction in particle diameter, assuming that the κ of the evaporating compound is identical to the κ of the particle residual (*44*). Simulations with a kinetic evaporation model (fig. S6) and assuming a 100% removal efficiency of the denuder suggest that the saturation vapor pressure of the evaporating compounds must exceed ~5 × 10^−5^ Pa if positive Δκ_rel_ is to be explained by a loss of mass of absorbed compounds. Plausible candidate compounds for evaporation are nitric acid dissolved in aqueous particles (particularly at higher RH) and semivolatile inorganic or organic compounds. Calculations estimating the maximum amount of nitric acid in aqueous droplets at RH = 50% (the upper limit of wet-selected particles) based on Henry’s law partitioning, Köhler theory (*7*, *45*) and an assumed maximum ambient gas-phase nitric acid concentration in urban air masses of 20 parts per billion (ppb) (*46*, *47*) suggest that the maximum Δκ_rel_ due to nitric acid is <5% (fig. S7). Typical average nitric acid measured by the CIMS between May and June was 0.4 ppb with maximum concentrations during that period peaking at ~4 ppb. Thus, the effect of nitric acid on Δκ_rel_ is even smaller. Similarly, an assumed maximum ambient gas-phase glyoxal concentration of 3 ppb (*48*, *49*) corresponds to a maximum Δκ_rel_ of <15% (fig. S7). Both are smaller than the observed Δκ_rel_. The saturation vapor pressure of subcooled ammonium nitrate at room temperature is ~4 × 10^−4^ Pa at 25°C and at least an order of magnitude lower for solid ammonium nitrate (*50*). The average mass fraction of nitrate ions measured by the aerosol mass spectrometer was ~0.05 for particles sized between 50 and 180 nm. Ammonium nitrate loss by volatilization plausibly contributed to some of the observed Δκ_rel_ but cannot account for the totality of the change. Regression of Δκ_rel_ with NO_3_^−^ fraction showed a negative slope (Fig. 2), which is consistent with nitrate volatilization. Furthermore, partitioning of semivolatile ammonium nitrate and organics due to an ~30% change in RH and an ~3°C change in temperature was directly observed during the Eastern Pacific Cloud Aerosol Precipitation Experiment (EPCAPE) by comparing measurements from the Scripps Pier and Mt. Soledad site (this work), which are 3 km apart (*51*). This directly confirms that semivolatile compounds were present in the aerosol sampled by the CCN instrument. Nevertheless, evaporation of absorbed compounds can only decrease the amount of dissolved solutes and in turn cause an increase in Δκ_rel_. It cannot explain an increase in κ with denuding, i.e., Δκ_rel_ < 0, unless one invokes that organic coatings provide a kinetic limitation to droplet activation, which has been disproved even for hydrophobic organic coatings by recent experiments (*52*, *53*).

Observed Δκ_rel_ < 0, measured at RH < ~30%, could be interpreted as an adjustment in interfacial tension between the droplet and air caused by insertion of the denuding step. Aqueous droplets have been shown to become quickly contaminated by adsorption of trace gases (*54*), which has been shown (*54*) or suggested by inference (*22*, *24*) to lower the droplet surface tension. Removing these gases from the aerosol may help desorb those compounds from the ~50- to 100-nm (hydrated and aqueous) particles or prevent further adsorption of trace gases as the particles grow by water uptake to >1000 nm to the point of droplet activation (*55*). The net effect of the adsorbed trace gas is to depress the formation of droplets (equivalent to raising interfacial tension) when particles are size selected at low RH (Δκ_rel_ < 0), which is opposite in direction to the earlier reports that adsorbed trace gases lower surface tension and enhance the formation of droplets. This is a puzzling result. Negative free energy of transfer from the bulk phase to the surface phase is needed for a compound to adsorb at the interface (*56*), which is usually obtained from a reduction in the interfacial tension. Gas adsorption coupled with raising interfacial tension requires an additional force. The presence of strong electric fields, exceeding 10^8^ V m^−1^ across aqueous interfaces (*57*), may drive electrostatically mediated adsorption of trace gases at the interface, which then could raise the interfacial tension. If interfacial electrostatic-aided trace gas adsorption indeed plays a role, it might also, at least in part, explain the dependence on RH. At higher RH, the droplet is more dilute, possibly altering the electric field strength and adsorption energy balance. This is indicative that thermodynamic and kinetic interactions of trace gases with the aqueous droplet are both important and not well understood. Sareen *et al.* (*22*) showed that the reduction in κ due to gas-phase methyl-glyoxal and acetaldehyde required exposure times of 5 hours, starting with dry ammonium sulfate particles. This time is much longer than the timescales considered here and indicates slow adsorption kinetics. There is a possibility that the silica gel dryer also acted as a denuder, at least for some trace gases. Silica gel is an adsorbent that acts through hydrogen bonds and may scrub some alcohols, amines, and other species (*58*). If true, the correlation between RH and the effect of denuding would be explained by a simultaneous exhaustion of the silica gel with respect to water vapor and trace gases. Detailed laboratory studies investigating the kinetics with dry and hydrated particles, as well as drying methods other than silica gel dryers will be needed to fully explain the field data.

The influence of semivolatile compounds on CCN activity may explain, at least in part, the discrepancy between subsaturated and supersaturated hygroscopicity measurements. Commonly cited explanations for the discrepancy are the potential mismatch of dry diameter measured by the HTDMA and the CCN at activation, the presence of dissolved water soluble gases in the droplet at high RH (*45*, *59*–*61*), surface active organics from the bulk solution lowering the interfacial tension at the air/droplet interface (*62*–*66*), adsorption of trace gases from the air lowering the interfacial tension of the air/liquid interface (*67*–*71*), liquid-liquid phase separation at high RH (*72*, *73*), gradual dissolution of sparingly soluble species as dilution increases with *RH* (*29*, *31*, *74*, *75*), nonspherical particle shape during mobility selection (*76*–*78*), or variations of κ with RH due to nonideal solution behavior (*31*). The normalized mean bias between CCN-derived κ and HTDMA-derived κ decreases from ~40 to ~13% when switching from undenuded to denuded CCN (fig. S11). However, the effect is limited to cases where inlet RH > 30% and may be a coincidence due to the reduction in CCN-derived κ from denuding at those humidities.

Nevertheless, the effect shown here is likely of critical importance for understanding aerosol-cloud interactions. There are several ways to estimate the magnitude of the effect. For example, global simulations by Liu and Wang (*79*) suggest that changing κ of the organic fraction by ±50% changes the CCN concentration within 40%. Calculations by Sareen *et al.* (*22*) for the response of stratus cloud droplet number concentrations to perturbations of 20% in κ suggest that the effect here would lead to a minimum change in cloud droplet number concentration of ~10%. For constant liquid water content, this corresponds to ~3% reduction in cloud effective radius and increase in cloud albedo by 0.8% (*22*). While the exact global influence on CCN and droplet number concentration will depend on the concentration and spatial distribution of the trace gas(es) responsible for the perturbation in Δκ_rel_, the effect is important enough to call into question whether current CCN measurements accurately reflect aerosol-cloud interactions in the atmosphere.

## MATERIALS AND METHODS

The instruments were colocated with the Department of Energy’s Atmospheric Radiation Measurement (ARM) program’s EPCAPE campaign. Measurements were made at two sites: the main site (M1) located at Scripps Pier in La Jolla, CA and an ancillary site (S2) atop Mt. Soledad (32.8398°N, 117.2523°W). The EPCAPE campaign took place over a 1-year period between 15 February 2023 and 14 February 2024.

### CCN measurements

The CCN instrument was housed inside a temperature-controlled trailer located at the S2 site. Measurements were performed between 9 February 2023 and 18 August 2023 (*80*). Aerosol size distributions and size-resolved CCN activity concentration was measured using a condensation particle counter (TSI 3772, 1 liter min^−1^ sample flow) and a CCNc (*9*) (Droplet Measurement Technologies Inc., Boulder CO, 0.5 liter min^−1^ sample flow) prefaced by a DMA (TSI 3080 long column, 5 liter min^−1^ recirculating sheath flow) operated in scanning mode. The setup was similar to that used in previous studies by our group (*13*, *81*, *82*). All aerosol sample lines were made of either stainless steel or copper tubing to prevent outgassing of organic compounds into the sample. Before sampling, particles were drawn through a counterflow virtual impactor during in-cloud periods or a total aerosol inlet (*83*) when cloud droplets were not present at the inlet. All data here are drawn from out-of-cloud periods. Particles were dried using a silica gel dryer and charge equilibrated using a ^210^Po neutralizer. RH and temperature were measured at the inlet of the instrument inside the trailer (Rotronic HC-2). A charcoal diffusion denuder (Delta Adsorbents, mesh size 4 mm) was inserted between the exit of the DMA and the inlet of the CCNc. Calculated transmission efficiencies suggest that the denuder removed molecules with fewer than 10 carbon atoms with >99% efficiency (fig. S1). Activated charcoal can be used for at least 3 months of continuous field operation without notable loss in gas-phase removal efficiency (*84*). Therefore the diffusion denuder was refilled with fresh adsorbent mid-campaign on 24 May 2023. The duty cycle of the instrument was as follows: First. the supersaturation is set in the CCN instrument. A 100-s hold is inserted to allow the temperature to equilibrate in the CCN instrument. Next, the voltage is ramped exponentially from 35 to 6000 V to 35 V over 240 s. After completion of the scan, an automated valve toggled between the undenunded branch (denuder bypassed) and the denuded branch. Last, the supersaturation was switched to the next value, sequentially stepping through supersaturation 0.2, 0.4, 0.6, 0.8, and 1%. The size distribution was reconstructed using a 0th order Thikonov inversion constrained by an initial guess (*85*). The CCN activation spectrum and activation diameter was obtained using the approach given in (*86*), which accounts for the effect of multiply charged particles. The κ was calculated from the instrument supersaturation and activation diameter (*7*). The instrument was calibrated using atomized, dried, and charge neutralized ammonium sulfate (*81*, *87*). The calibration showed no difference in inferred supersaturation between the denuded and undenuded branches. A schematic of the setup, further details about the duty cycle, and calibration results are in the Supplementary Materials.

### HTDMA measurements

A HTDMA (model 3100, Brechtel Manufacturing Inc., Hayward, CA, USA) was operated by the ARM program and housed at the M1 site (*88*). The HTDMA selects a dry diameter, humidifies the monodisperse sample flow to RH = 85%, and measures the wet size distribution in the second DMA. The system cycled through dry diameter set points of 50, 100, 150, 200, and 250 nm. Data inversion accounting for the DMA transfer function and multiply charged particles is based on regularization (*89*) and is identical to the description in section 2.2.3 in Kasparoglu *et al.* (*15*). Briefly, this method stipulates two populations with growth factors *g*_1_ and *g*_2_, a more and less hygroscopic mode. The κ of these modes were calculated from *g*, set point humidity, and dry diameter (*7*). Here, the more and less hygroscopic modes fitted were strongly correlated, close in growth factor, and had relative fractions of ~0.5. Thus, the interpretation of the hygroscopicity of the more and less hygroscopic modes represent a lower and upper bound of the range of hygroscopicity values of an externally mixed population.

### CIMS measurements

The iodide-CIMS used during this campaign has been previously described in Hayden *et al.* (*90*) and more recently in Young *et al.* (*91*). There were two important modifications to the instrument from its operation in Young *et al.* (*91*). First, the radioactive source (NRD P-2021) was replaced by a vacuum ultraviolet (VUV) source (Restek Photoionization Lamp, Model 108-BTEX), in a similar manner to other groups (*92*–*94*). The second change was that of the source gas. To use the typical methyl iodide (CH_3_I) permeation oven source (regulated to 40°C) with nitrogen (N_2_) carrier gas, it was necessary to add a benzene (C_6_H_6_) permeation oven (regulated to 70°C) in series before the gas flow reached the lamp. The benzene vapor acted as a source of photoelectrons to amplify the amount of I^−^ produced in the ion source. The VUV source requires a flow of only 250 standard cubic centimeter per minute (SCCM) (versus ~2 sLpm) of N_2_ to generate reagent ion count rates comparable to that of the previous ^210^Po source. The instrument was calibrated in situ through the addition of isotopically labeled (^13^C) propanoic acid in a flow of 50 SCCM of N_2_ to the inlet. All other calibrations were performed postcampaign using a combination of permeation tubes, calibration cylinders, and liquid calibrations.

### Back trajectory clustering

In this study, 48-hour ensemble-mean back trajectories (ARMTRAJ) were used for Mt. Soledad (*95*). Surface air mass trajectories (ARMTRAJSFC) were retrieved for every 3 hours (UTC) originating at 0 to 50 m above mean sea level using the Hybrid Single-Particle Lagrangian Integrated Trajectory model at 0.25° and 1-hour spatial and temporal resolutions with the fifth-generation European Centre for Medium-Range Weather Forecasts atmospheric reanalysis (ERA5) (*96*). At Mt. Soledad, ARMTRAJSFC was used when Mt. Soledad was within the planetary boundary layer (PBL) height derived using the Richardson method. Free troposphere back trajectories (ARMTRAJPBL) are used when Mt. Soledad is above the PBL height. The trajectories were categorized using a *k*-means clustering algorithm to identify unique mean trajectories during EPCAPE to represent the origin of air masses (*97*). Five different clusters of back trajectories were identified and are termed Coastal North-Westerly, Los Angeles–Long Beach, Southerly, Easterly, and Marine Westerly (*98*).

### AMS measurements

A high-resolution time-of-flight aerosol mass spectrometer (HR-ToF-AMS, Aerodyne Research Inc., Billerica, MA) was deployed at the S2 site for the entirety of EPCAPE and housed in the same trailer as the CCN instrument. During cloud-free periods, the HR-ToF-AMS sampled behind a PM_1_ cyclone located after the main isokinetic aerosol inlet. Mass spectra measurements under V-mode provided the submicrometer mass concentrations of nonrefractory inorganic (sulfate, nitrate, ammonium, and chloride) and organic components every 5 min. In this study, mass concentrations of nonrefractory components were estimated for the 50- to 180-nm size range using size-resolved mass concentration data from HR-ToF-AMS.

## Supplementary Materials

### This PDF file includes:

Supplementary Text S1 to S7 Figs. S1 to S15 References
