# Supplementary material for: Removal of trace gases can both increase and decrease cloud droplet formation
Source: Sci Adv. 2026 Jan 14;12(3):eadx0960. doi: 10.1126/sciadv.adx0960 (PMC12802841; doi:10.1126/sciadv.adx0960)
Supplement: Supplementary file 1 — Supplementary Text S1 to S7 Figs. S1 to S15 References [file sciadv.adx0960_sm.pdf]

Science Advances
AAAS

Supplementary Materials for

Removal of trace gases can both increase and decrease cloud droplet formation

Elavarasi Ravichandran *et al.*

Corresponding author: Markus D. Petters, markus.petters@ucr.edu

Sci. Adv. **12**, eadx0960 (2026)
DOI: 10.1126/sciadv.adx0960

This PDF file includes:

Supplementary Text S1 to S7
Figs. S1 to S15
References

S1. Denuder

The denuder consists of two repurposed diffusion driers (TSI Model 3062) in series. The drier is a cylindrical plexiglass tube ($D = 0.076\,m$, $L = 0.403\,m$). Inside is a mesh ($D = 1.3\,cm$) that runs the entire length of the tube. Activated charcoal (Delta Adsorbents, mesh size 4mm) fills the space between the mesh and the plexiglass. Activated charcoal was used because it maintains its effectiveness for at least 3 months (*84*). Sample flows at volumetric flow rate $Q = 0.5\,L\,min^{-1}$ through the inside of the mesh. The theoretical collection efficiency (CE) in a cylindrical diffusion denuder is be calculated via

$$\frac{C}{C_0} = 0.8191 \times exp(14.6272 \times \Delta) + 0.0976 \times exp(89.22 \times \Delta) + 0.01896 \times exp(-212 \times \Delta) \quad \text{(S1.1)}$$

where $\Delta = (\pi DL)/(4Q)$ is a dimensionless parameter, L is the denuder length, D is the diffusion coefficient, Q is the air flow rate, C_0 is the analyte concentration at the denuder inlet and C is the mean analyte concentration at the outlet. The CE is then defined as $CE = 1 - C/C_0$. For fixed flow rate, the CE only depends on the diffusion coefficient of the material.

To illustrate the type of Volatile Organic Compounds (VOC) collected, the diffusion coefficient of a select number of VOCs is evaluated using a mix of observational data (*101*) and prediction from functional group data. Prediction of diffusion coefficient from kinetic theory and functional group data is performed following Eq. 17.3-12 from Bird et al. (2002) (*102*). The collision cross-section of the molecule can be estimated from the critical volume using the Lyderson group contribution method (*102*, *103*). Melting points can be used to estimate Lennard-Jones parameters for a given material. The collision integral is determined using Eq. E2-2 from Bird et al. (2002) (*102*). Melting points were obtained from the National library of medicine (*104*).

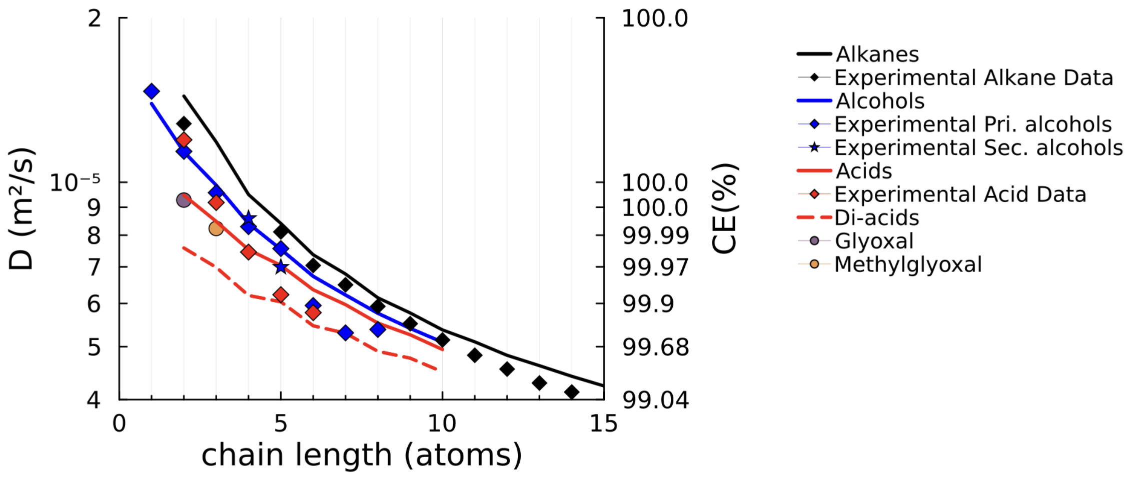

Figure S1. Diffusion coefficient estimates for alkanes, alcohols, acids, glyoxal and methylglyoxal, as a function of carbon chain length. Lines: diffusion coefficient calculated from kinetic theory. Symbols: experimental data except for glyoxal and methylglyoxal which are estimated using kinetic theory. The right axis shows the calculated collection efficiency calculated using Eq. (S1.1).

Figure S1 summarizes the diffusion coefficient and collection efficiency for common alkanes, alcohols, aldehydes, and acids as a function of carbon chain length. Larger carbon chain lengths have lower diffusion coefficients and lower collection efficiency. The removal efficiency reaches about 100% when the diffusion coefficient exceeds 9×10^{-6} m^2/s. Furthermore, the collection efficiency exceeds 99% for all compounds considered. Thus the denuder removes alkanes, and weakly functionalized acids, aldehydes and alcohols with fewer than 10 carbon atoms with > 99% efficiency from the gas phase.

S2. Instrument Setup and Duty Cycle

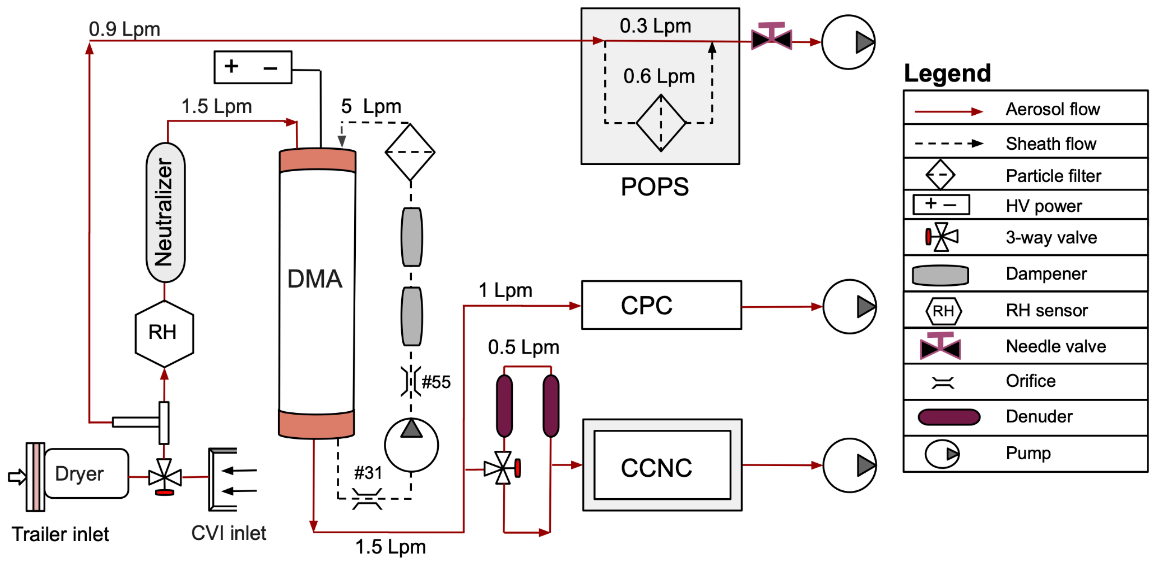

Figure S2. Schematic of the instrument setup.

Figure S2 shows a schematic of the instrument setup inside the trailer. Details are in the main text. Figure S3 shows the voltage and supersaturation profiles during a Cloud Condensation Nuclei (CCN) instrument's cyclic operation, in which the system alternates between denuded and undenuded conditions. This cycle is intended to study how denuding, or the removal of volatile components, affects a particle's ability to serve as a CCN. The voltage trace demonstrates a repeating pattern over a 5-minute cycle, where the system alternates between two states: denuded and undenuded. An initial 100-second hold is followed by a 120-second voltage increase from 35 V to 6000 V in each cycle. Following a 10-second hold at 6000 V, the voltage drops to 35 V over the course of another 120 seconds, ending with a final 10-second flush. The instrument alternates between the denuded and undenuded states in a systematic way to sample each state under the same conditions, repeating this voltage pattern. In parallel, the CCN instrument's supersaturation (*S*) varies gradually, beginning at 0.2%, increasing by 0.2% to 1%, and then dropping back to 0.2%. The instrument's applied thermal gradients (dT) correlate to this regulated change in supersaturation.

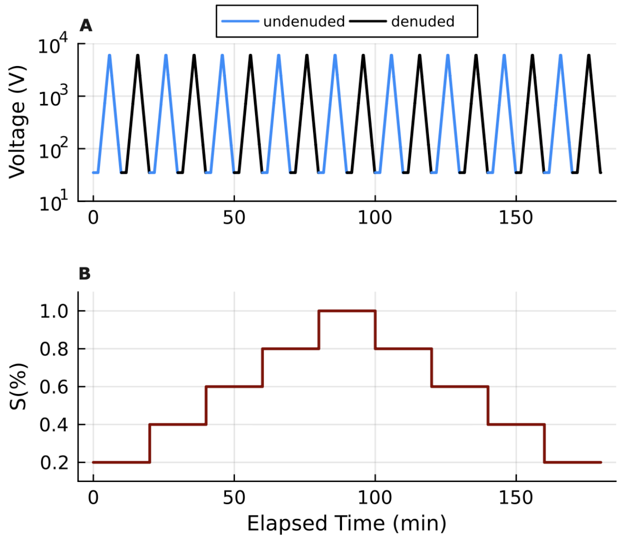

Figure S3. Voltage and supersaturation profiles during a 180-minute period of cyclic denuded and undenuded operation of the CCN instrument. (A) The alternating voltage scans between the denuded (black) and undenuded (blue) states. Each cycle lasts for five minutes. **(B)** The corresponding supersaturation values (*S*), which gradually rise from 0.2% to 1% and then fall back to 0.2%.

Water supersaturation inside the CCN was calibrated prior to the start of the campaign. Dried charge-neutralized ammonium sulfate particles were passed to the denuded CCN system. The CCN counter was operated at fixed thermal gradients dT. The activation diameter was obtained from the activation diameter fitted to the curve of particle dry diameter vs. the ratio of CCN and CN concentration (*86*, *105*). The activation dry diameter was converted to supersaturation using the E-AIM model (*106*) and the equation in Section 4.1 of Petters and Petters (2016) (*81*). Figure S4 summarizes the supersaturation calibration applied during the campaign. A linear fit between supersaturation (s_c) and thermal gradient (dT) was used due to the expectation of a linear relationship from a model of the instrument (*9*, *81*). Gradients corresponding to S = 0.2, 0.4, 0.6, 0.8, and 1% were calculated from the fit and used throughout the campaign. Figure S4 also shows that there is no systematic difference in supersaturation between the denuded and undenuded branch of the measurement for laboratory generated inorganic particles. No post campaign calibration was performed due to failure of the condensation particle counter and the need to ship the instruments to the manufacturer for refurbishing. However, the calibration in DMT CCN is not prone to drift (*107*). Furthermore, the droplet diameter measured at the exit of

the CCNc is proportional to the supersaturation. A drift in supersaturation over time would therefore correspond to drift in mean diameter. Figure S5 summarizes the observed mean droplet diameter throughout the campaign. The data show no systematic change for any of the supersaturations used during the study.

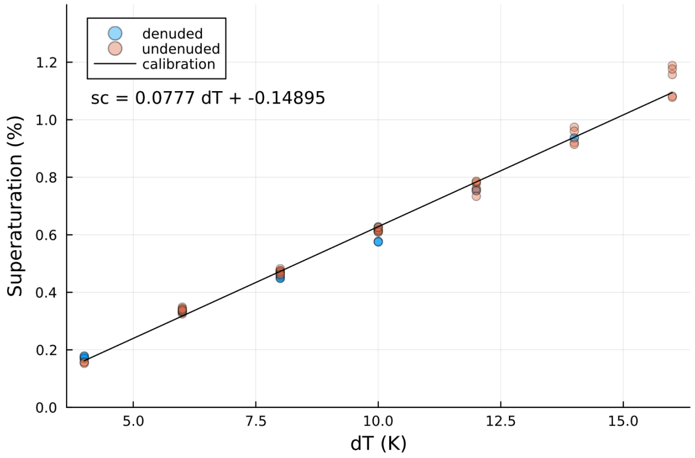

Figure S4. Supersaturation calibration obtained prior to the start of the campaign.

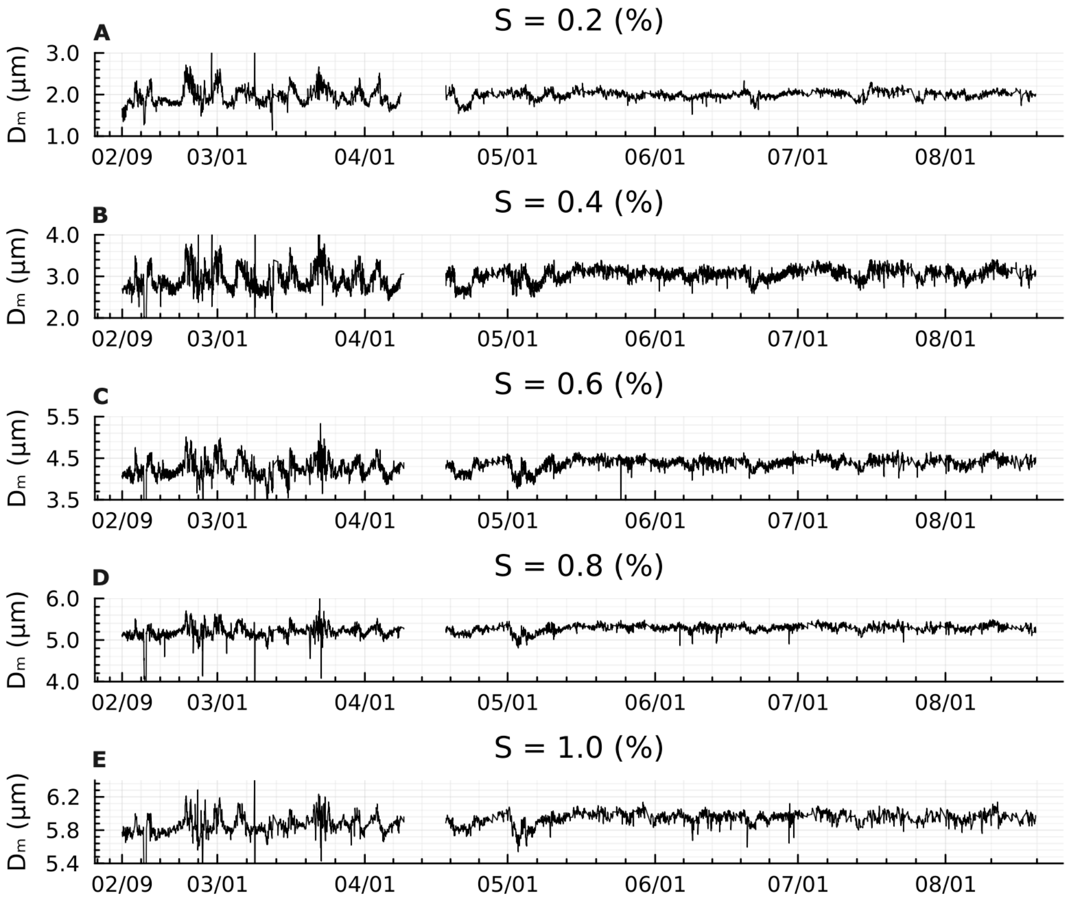

Figure S5. (A - E) Mean mode diameters for the campaign across all supersaturation levels. Missing data in April is due to instrument malfunction.

S3. Evaporation Model

To evaluate the hypothesis that mass loss by evaporation can account for a decrease in κ after passage through the denuder, the following evaporation model is used (*108*)**:**

$$\frac{dD_p}{dt} = -\frac{4DM}{\rho D_p RT} p^0 \, exp\left(\frac{4\sigma M}{D_p \rho RT}\right) F(Kn, \alpha) \quad \text{(S1.2)}$$

where D is the diffusivity, M is the molar mass, D_p is the particle diameter, R is the gas constant, ρ is the density, T is the temperature, σ is the surface free energy, p^0 is the vapor pressure over a flat surface and $F(Kn, \alpha)$ is a correction term Eq. 3 from Bilde et al., (2003) (*108*) where $Kn = 2\lambda D_p$ is the Knudsen number and λ is the mean free path, calculated as $\lambda = 3D/c$ and $c = \sqrt{(8RT)/(\pi M)}$. The equation assumes that the vapor pressure in the gas phase is zero.

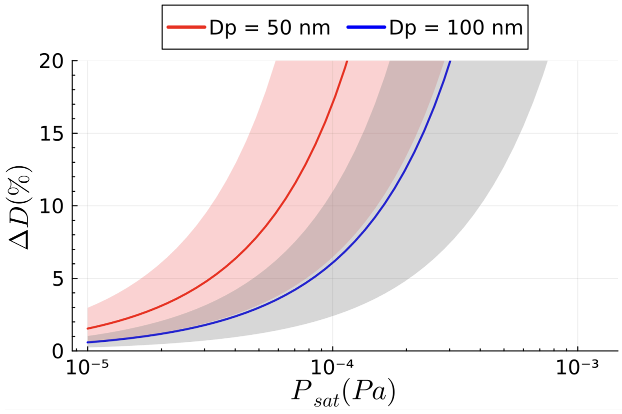

Figure S6. Percentage change in diameter after 20 s evaporation for initial diameters of 50 nm and 100 nm vs. saturation vapor pressure. The solid red and blue lines assume a molar mass of 100 g/mol. The shading indicates variability based on a range of molar masses between 30 and 200 g/mol.

The denuder adds an extra 20 s of residence time between the exit of the DMA and detection by the CPC (Figure S2). Therefore, Eq. (S1.2) is integrated over $t = 0$ to 20 s. Figure S6 shows the corresponding diameter change as a function of vapor pressure for initial particle diameters of 50 nm and 100 nm. The assumed diffusion coefficient used is 7×10^{-6} m^2/s based on typical values summarized in Figure S1. However, the net result is insensitive to the assumed value due

to the inclusion of the $F(Kn_i, \alpha_i)$ term. The result is, however, sensitive to the assumed molar mass and this sensitivity is illustrated by the shading. The sensitivity of κ to a change in D_p is $\partial \ln \kappa / \partial \ln D_P = 3$(*44*), i.e. a 10% change in size corresponds to a 30% change in κ. Thus, to explain a 30% change in κ by evaporation of particle mass after passage through the denuder, the saturation vapor pressure of the compounds would have to range between 3×10^{-5} Pa and $>3\times10^{-4}$ Pa. Furthermore, these compounds must have been present at saturation ratios close to unity prior to passage through the denuder to prevent evaporation in the undenuded branch.

S4. Sensitivity of κ to water soluble trace gases

The saturation ratio of water over a solution droplet, S_w, is given by (*7*, *45*)

$$S_W = \frac{D^3 - D_d^3}{D^3 - D_d^3(1-\kappa)} exp\left(\frac{4\sigma_{s/a} M_w}{RT\rho_w D}\right) - \nu_a (P_a K_a)^{1/2} \quad \text{(S1.3)}$$

Where D is the diameter of the droplet, D_d is the dry diameter, κ is the hygroscopicity parameter, $\sigma_{s/a}$ is the surface tension of the solution, M_w is the molecular weight of water, R is the universal gas constant, T is temperature, ρ_w is the density of water, ν_a is the stoichiometric coefficients of the individual ions, P_a is the partial pressure of the solute and K_a is Henry's law coefficient in units of atm^{-1}. The relative humidity is $RH = 100S_w$. Eq. (S1.3) is the same as Eq. 23 in Laaksonen et al. (*45*) but the approximate expression for traditional Kohler theory in their work is replaced with the κ-Kohler equation (*7*). The relative error Δκ is calculated by evaluating D for a fixed S_w according to Eq. (1.3) and then deriving the effective κ from the calculated D and S_w using the standard approach, i.e. without the trace gas partitioning term. The resulting Δκ expresses the potential influence of the trace gas on the observed κ.

Here nitric acid and glyoxal are examined as a representative inorganic and organic trace gas. Values obtained from the literature were converted from $\frac{mol}{m^3 Pa}$ to atm^{-1} using the specified conversion factor 1.831 (*109*, *110*). The reported values for Henry's constant varied widely. The midpoint between the upper and lower bounds of reported Henry's constant values were calculated to obtain a representative value. The calculated Henry's law constant midpoint for nitric acid and glyoxal are $1.41 \times 10^4 \frac{mol}{m^3 Pa}$ and $2.47 \times 10^5 \frac{mol}{m^3 Pa}$ which corresponds to $2.57 \times 10^4\ atm^{-1}$ and $4.52 \times 10^5\ atm^{-1}$ respectively (*109*, *110*). The effect of nitric acid on the change in hygroscopicity of a particle with $\kappa = 0.3$ at 50% RH was evaluated over a concentration range of 1 to 100 ppb, with 20 ppb reflecting typical ambient levels in urban environments (*46*, *47*). Similarly, glyoxal concentrations were assessed between 1 and 10 ppb, with approximately 3 ppb representing typical urban ambient concentrations (*48*, *49*). Figure S7 shows a clear enhancement in the hygroscopicity parameter (positive Δκ) with increasing

concentrations of both nitric acid (HNO_3) and glyoxal at RH = 50%, although the magnitude and response differ between the two compounds. At 100 ppb of nitric acid, the mean Δκ is approximately 20%, indicating a steady increase in hygroscopicity as nitric acid levels rise. However, glyoxal exhibits a more pronounced effect, with the mean Δκ reaching around 30% at just 10 ppb of glyoxal, suggesting a stronger impact on particle hygroscopicity relative to HNO_3. The red shaded regions represent the uncertainty in Δκ values, which arise due to variability in the Henry's constant values used in the calculations. These findings underscore the stronger relative influence of glyoxal on particle hygroscopicity at lower concentrations compared to nitric acid, while highlighting the variability introduced by uncertainties in fundamental parameters like Henry’s constant.

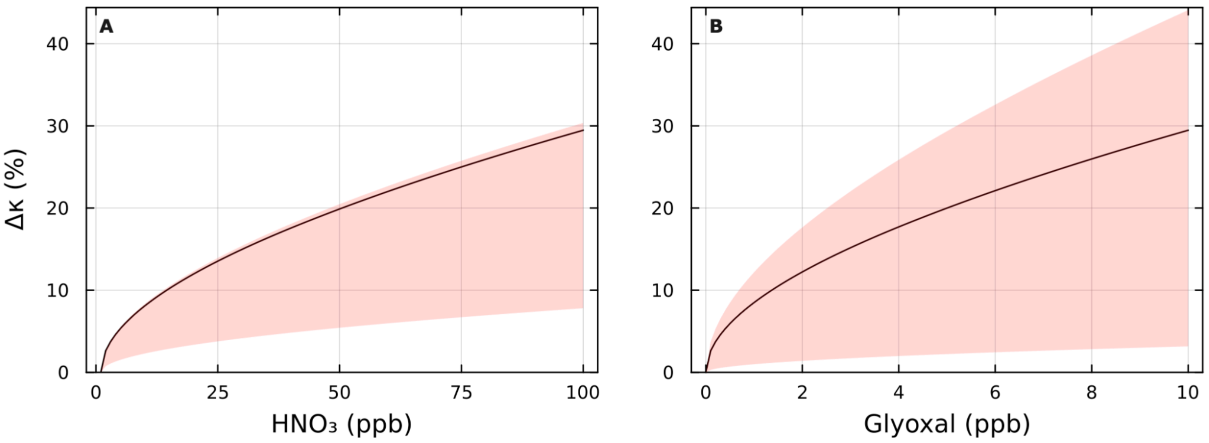

Figure S7. Percentage change in hygroscopicity parameter (Δκ). (A) The variation of κ with respect to nitric acid (HNO_3) and **(B)** glyoxal gas-phase concentrations at RH = 50%, assuming an initial value of κ = 0.3. The black lines represent the mean Δκ values, while the red shaded regions represent the uncertainty in Δκ due to the variation in Henry's constant values.

S5. HTDMA-derived κ vs. CCN-derived κ

Figure S8 shows an example inversion (*85*, *111*) of HTDMA data for 50 nm dry particles measured during the campaign. The inversion assumes populations with growth factors g_1 and g_2 and relative fraction f of the more hygroscopic mode. The parameters $g_{1,}$ g_2, and f are found by calculating the forward transmission through the instrument and minimizing the residual between the modeled and observed growth factor distribution. Although two modes were needed to fit the data, termed the more and less hygroscopic mode, the growth factor distributions generally exhibited an effective monomodal shape. Furthermore $g_{1,}$ g_2 are strongly correlated and f does not strongly vary for the duration of the campaign. We thus treat the more and less hygroscopic growth factor (and associated hygroscopicity parameter) as upper and lower bounds encapsulating the growth factor distribution. The most probable growth factor is calculated as the fraction weighted mean of the two modes. The HTDMA calibration at the start and end of the campaign showed no notable differences.

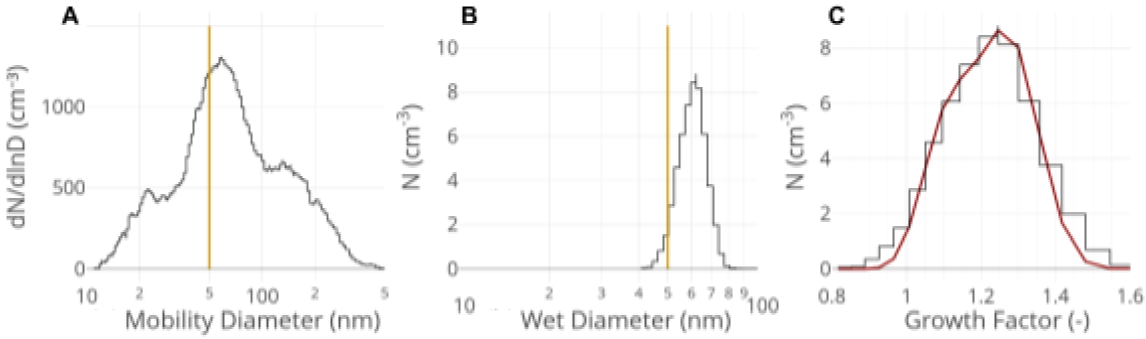

Figure S8. Example inversion of 50 nm dry (golden lines) particles measured by the HTDMA 16 April 2023. (A) Aerosol size distribution from the colocated scanning mobility particle sizer, **(B)** humidified size distribution, **(C)** humidified size distribution expressed in growth factor units (black). The red line shows the best fit forward model assuming g_1 = 1.11, g_2 = 1.27, and a fraction f = 59% of the more hygroscopic mode. Sample RH = 85%.

Figure S9 shows the temporal evolution of the hygroscopicity from the CCN at Mt. Soledad, alongside the more and less hygroscopic modes from HTDMA collected at the Scripps pier. The data show that the CCN-derived κ (κ_{CCN}) correlates with the HTDMA-derived κ (κ_{HTDMA}) . Figure S10 shows κ_{HTDMA} and κ_{CCN} as a function of diameter. Both figures clearly show that κ_{CCN}

exceeds κ_{HTDMA}. Figure S11 shows scatter plots of κ_{CCN} and κ_{HTDMA} stratified by relative humidity and stratified by denuded and undenuded measurements. The normalized mean bias between κ_{CCN} and κ_{HTDMA} is ~40% for the undenuded measurements. Denuding at elevated relative humidity reduced κ_{CCN}, lowering the normalized mean bias to ~13%.

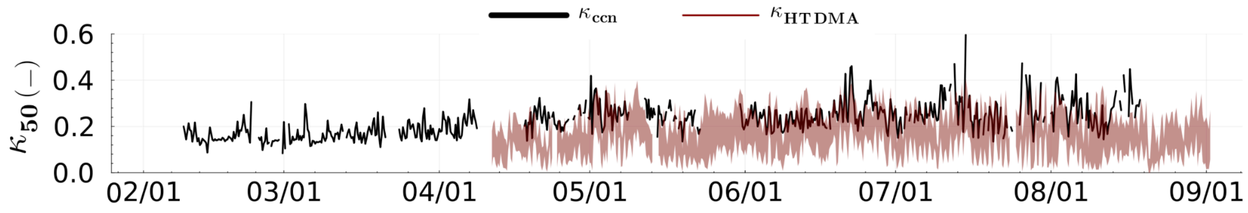

Figure S9. Comparison of the hygroscopicity parameter (κ) for 50 nm particles. κ derived from AOS HTDMA at M1 and CCNc at Mt. Soledad (red). The shading represents the κ of the less and more hygroscopic mode determined by the inversion.

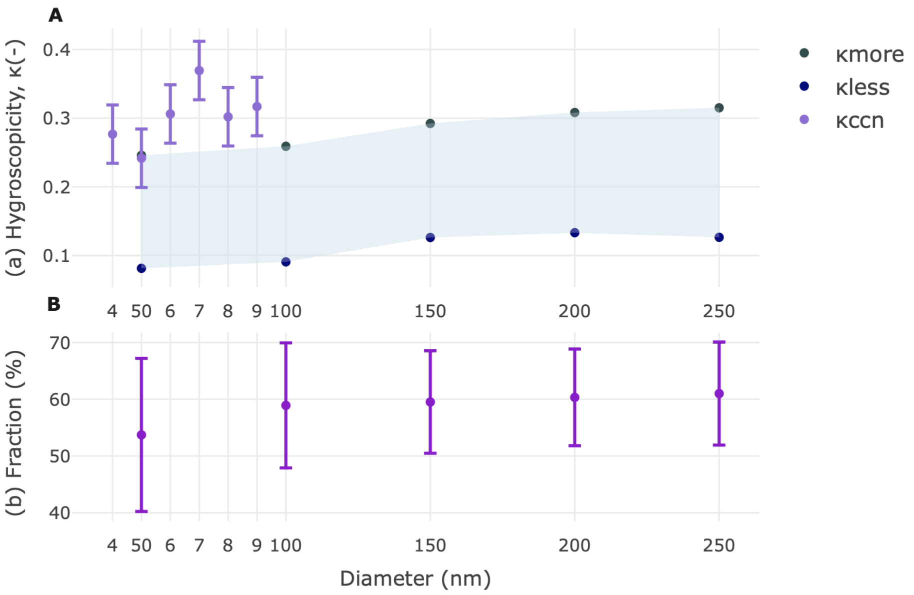

Figure S10. (A) Size dependence of the hygroscopicity of the more (grey) and less hygroscopic (navy) modes from the HTDMA and hygroscopicity of the CCN (purple) and **(B)** number fraction of the more hygroscopic mode (violet) during the intensive operating period. Points denote the mean and error bars the standard deviation.

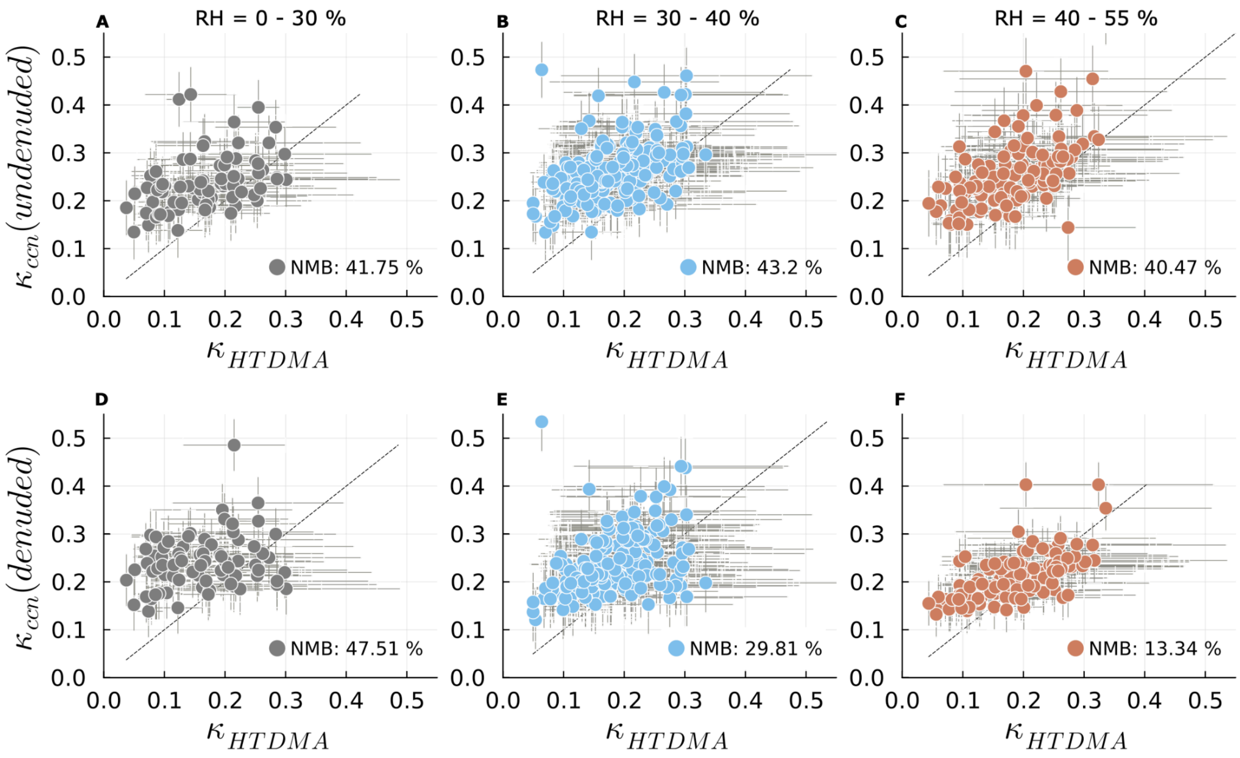

Figure S11. **(A-C)** Comparison of κ_{HTDMA} and κ_{CCN} for denuded and **(D-F)** undenuded measurements stratified by ranges of relative humidity. The normalized mean bias (NMB) is indicated in the legend.

S6. Regression Analysis

Figure S12 shows the correlation of $\Delta\kappa_{rel}$ with relative humidity for the period May 31 to July 6, 2023. This time period was selected to the largest contiguous segment where $\Delta\kappa_{rel}$, CIMS, and AMS data were available.

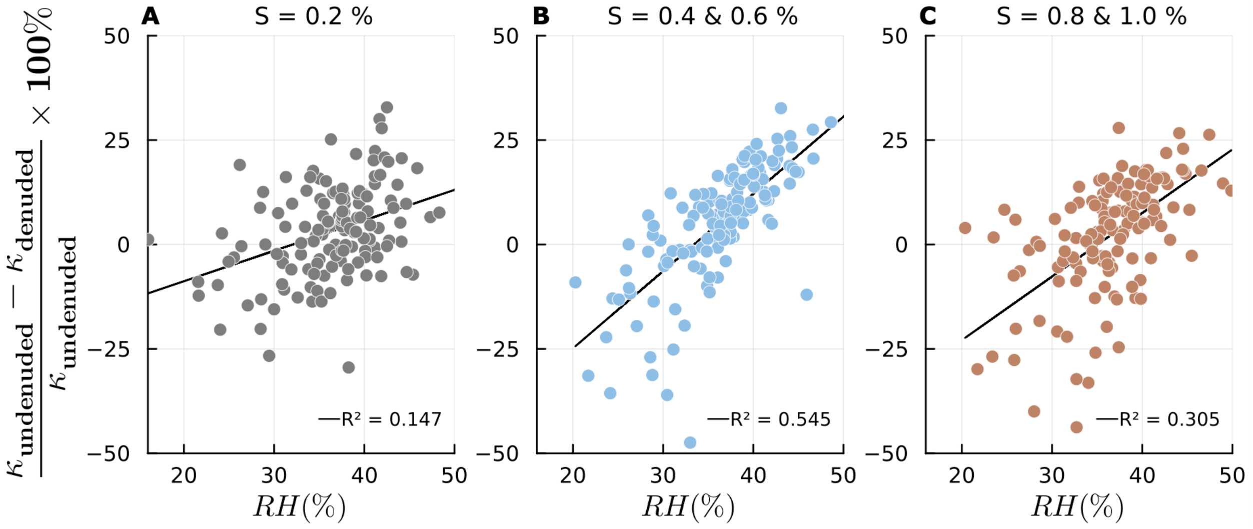

Figure S12. Correlation between the $\Delta\kappa_{rel}$ and RH. (A) Correlation between the relative percentage difference between undenuded and denuded κ and RH at $S = 0.2\%$, **(B)** 0.4% and 0.6%, and **(C)** 0.8% and 1.0%, from May 31 to July 6, 2023, averaged every 6 hours.

S7. Effect of denuding on CCN-derived κ

Figures S14 and S15 are the same as the main text but for supersaturations $S = 0.2$ and $S = 0.8$ and 1%.

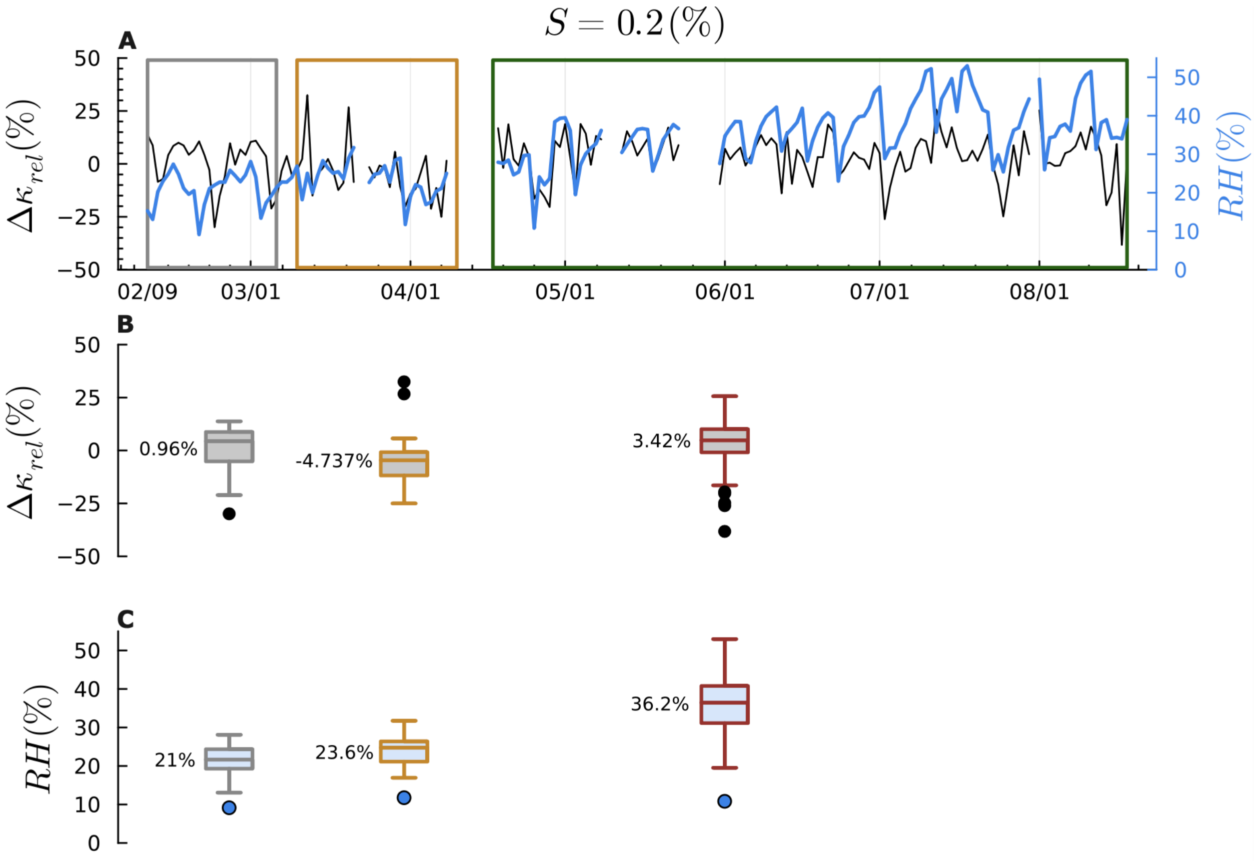

Figure S13. Same as Figure 1 (Main Text) but for *S* = 0.2%.

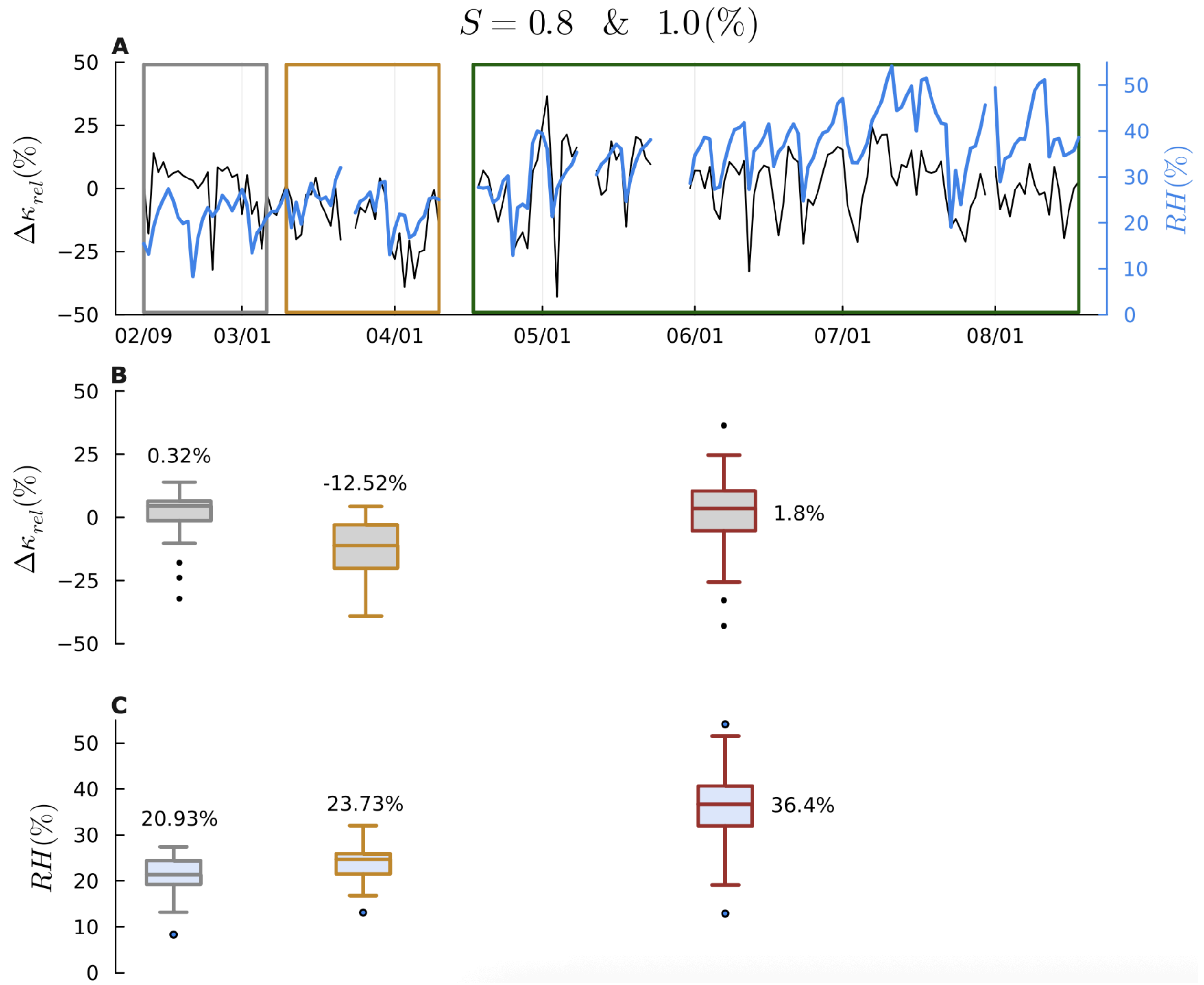

Figure S14. Same as Figure 1 (Main Text) but for *S* = 0.8 and 1%

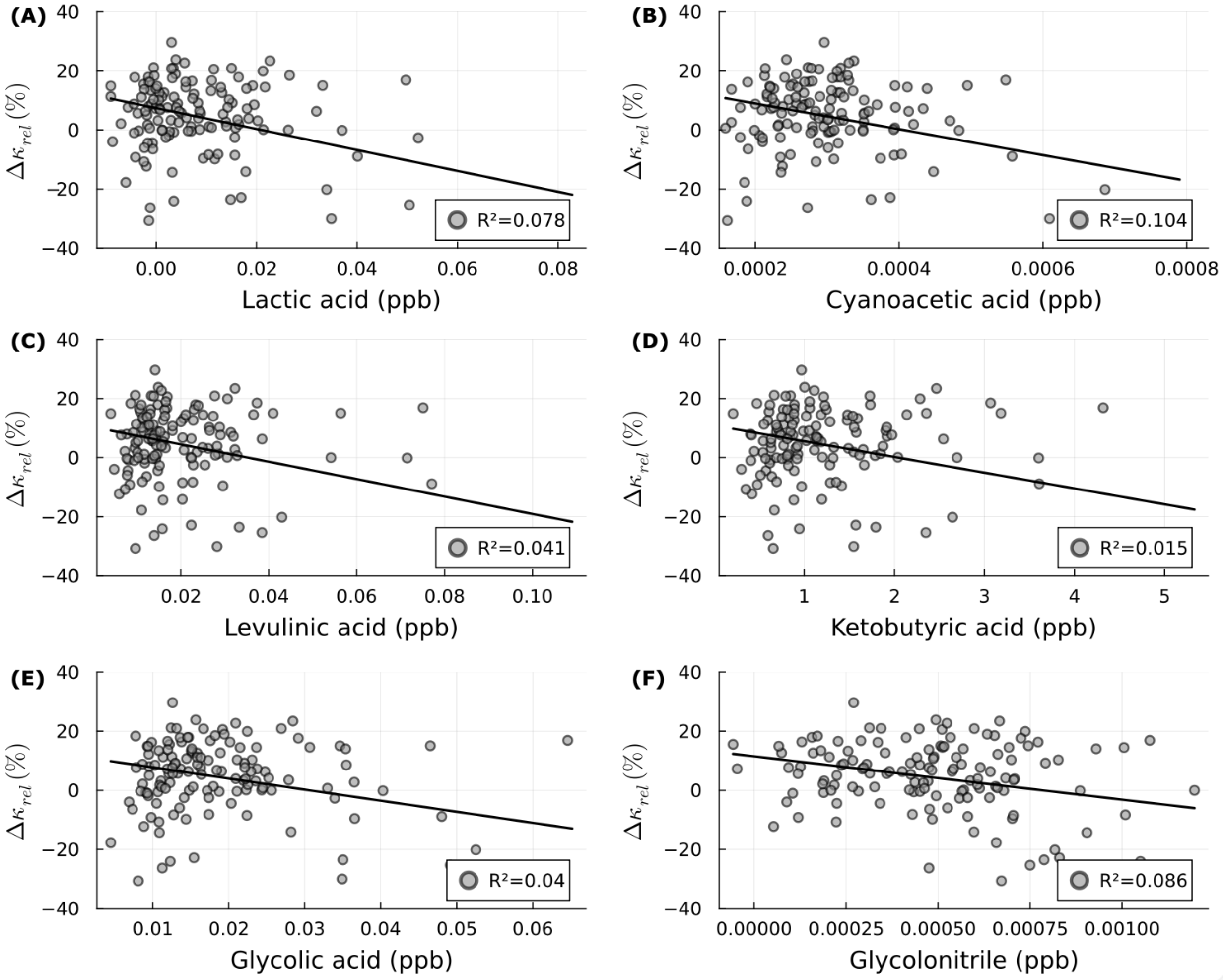

Figure S15. The relationship between the concentration (ppb) of top six compounds and $\Delta\kappa_{rel}$. The black line represents the predicted $\Delta\kappa_{rel}$ from a multiple linear regression including both compounds and RH. The R^2 value from the simple linear regression (compound vs. $\Delta\kappa_{rel}$) is indicated in the legend. **(A)** Lactic acid **(B)** Cyanoacetic acid **(C)** Levulinic acid **(D)** Ketobutyric acid **(E)** Glycolic acid **(F)** Glycolonitrile.

REFERENCES

1. S. Twomey, The influence of pollution on the shortwave albedo of clouds. *J. Atmospheric Sci.* **34**, 1149–1152 (1977).

2. B. Stevens, G. Feingold, Untangling aerosol effects on clouds and precipitation in a buffered system. *Nature* **461**, 607–613 (2009).

3. M. D. Petters, J. R. Snider, B. Stevens, G. Vali, I. Faloona, L. M. Russell, Accumulation mode aerosol, pockets of open cells, and particle nucleation in the remote subtropical Pacific marine boundary layer. *J. Geophys. Res. Atmospheres* **111**, doi.org/10.1029/2004JD005694 (2006).

4. J. Mülmenstädt, G. Feingold, The radiative forcing of aerosol–cloud interactions in liquid clouds: Wrestling and embracing uncertainty. *Curr. Clim. Change Rep.* **4**, 23–40 (2018).

5. T. Stevenson, J. Latham, K. Bower, T. Choularton, H. Coe, P. Connolly, G. Cooper, T. Craft, J. Foster, A. Gadian, L. Galbraith, H. Iacovides, D. Johnston, B. Launder, B. Leslie, J. Meyer, A. Neukermans, B. Ormond, B. Parkes, P. J. R. Rush, S. Salter, H. Wang, Q. Wang, R. Wood, Marine cloud brightening. *Philos. Trans. R. Soc. Math. Phys. Eng. Sci.* **370**, 4217–4262 (2012).

6. G. Feingold, V. P. Ghate, L. M. Russell, P. Blossey, W. Cantrell, M. W. Christensen, M. S. Diamond, A. Gettelman, F. Glassmeier, E. Gryspeerdt, J. Haywood, F. Hoffmann, C. M. Kaul, M. Lebsock, A. C. McComiskey, D. T. McCoy, Y. Ming, J. Mülmenstädt, A. Possner, P. Prabhakaran, P. K. Quinn, K. S. Schmidt, R. A. Shaw, C. E. Singer, A. Sorooshian, V. Toll, J. S. Wan, R. Wood, F. Yang, J. Zhang, X. Zheng, Physical science research needed to evaluate the viability and risks of marine cloud brightening. *Sci. Adv.* **10**, eadi8594 (2024).

7. M. D. Petters, S. M. Kreidenweis, A single parameter representation of hygroscopic growth and cloud condensation nucleus activity. *Atmos Chem Phys* **7**, 1961–1971 (2007).

8. S. M. Kreidenweis, M. Petters, U. Lohmann, 100 years of progress in cloud physics, aerosols, and aerosol chemistry research. *Meteorol. Monogr.* **59**, 11.1–11.72 (2019).

9. G. C. Roberts, A. Nenes, A continuous-flow streamwise thermal-gradient CCN chamber for atmospheric measurements. *Aerosol Sci. Technol.* **39**, 206–221 (2005).

10. J. R. Snider, M. D. Petters, P. Wechsler, P. S. K. Liu, Supersaturation in the Wyoming CCN Instrument. *J. Atmospheric Ocean. Technol.* **23**, 1323–1339 (2006).

11. J. G. Hudson, Cloud condensation nuclei measurements within clouds. *J. Clim. Appl. Meteorol.* **23**, 42–51 (1984).

12. K. J. Sanchez, L. M. Russell, R. L. Modini, A. A. Frossard, L. Ahlm, C. E. Corrigan, G. C. Roberts, L. N. Hawkins, J. C. Schroder, A. K. Bertram, R. Zhao, A. K. Y. Lee, J. J. Lin, A. Nenes, Z. Wang, A. Wonaschütz, A. Sorooshian, K. J. Noone, H. Jonsson, D. Toom, A. M. Macdonald, W. R. Leaitch, J. H. Seinfeld, Meteorological and aerosol effects on marine cloud microphysical properties. *J. Geophys. Res. Atmospheres* **121**, 4142–4161 (2016).

13. Y. Duan, M. D. Petters, A. P. Barros, Understanding aerosol–cloud interactions through modeling the development of orographic cumulus congestus during IPHEx. *Atmos Chem Phys* **19**, 1413–1437 (2019).

14. D. Rothenberg, C. Wang, Metamodeling of droplet activation for global climate models. *J. Atmospheric Sci.* **73**, 1255–1272 (2016).

15. S. Kasparoglu, N. Meskhidze, M. D. Petters, Aerosol mixing state, new particle formation, and cloud droplet number concentration in an urban environment. *Sci. Total Environ.* **951**, 175307 (2024).

16. S. Nakao, S. R. Suda, M. Camp, M. D. Petters, S. M. Kreidenweis, Droplet activation of wet particles: Development of the Wet CCN approach. *Atmos Meas Tech* **7**, 2227–2241 (2014).

17. D. O. Topping, G. McFiggans, Tight coupling of particle size, number and composition in atmospheric cloud droplet activation. *Atmos Chem. Phys.* **12**, 3253–3260 (2012).

18. T. K. V. Nguyen, M. D. Petters, S. R. Suda, H. Guo, R. J. Weber, A. G. Carlton, Trends in particle-phase liquid water during the Southern Oxidant and Aerosol Study. *Atmospheric Chem. Phys.* **14**, 10911–10930 (2014).

19. H. Herrmann, T. Schaefer, A. Tilgner, S. A. Styler, C. Weller, M. Teich, T. Otto, Tropospheric aqueous-phase chemistry: Kinetics, mechanisms, and its coupling to a changing gas phase. *Chem. Rev.* **115**, 4259–4334 (2015).

20. M. Kulmala, A. Laaksonen, P. Korhonen, T. Vesala, T. Ahonen, J. C. Barrett, The effect of atmospheric nitric acid vapor on cloud condensation nucleus activation. *J. Geophys. Res. Atmospheres* **98**, 22949–22958 (1993).

21. M. Kulmala, A. Laaksonen, R. J. Charlson, P. Korhonen, Clouds without supersaturation. *Nature* **388**, 336–337 (1997).

22. N. Sareen, A. N. Schwier, T. L. Lathem, A. Nenes, V. F. McNeill, Surfactants from the gas phase may promote cloud droplet formation. *Proc. Natl. Acad. Sci. U. S. A.* **110**, 2723–2728 (2013).

23. D. Topping, P. Connolly, G. McFiggans, Cloud droplet number enhanced by co-condensation of organic vapours. *Nat. Geosci.* **6**, 443–446 (2013).

24. A. E. Vizenor, A. A. Asa-Awuku, Gas-phase kinetics modifies the CCN activity of a biogenic SOA. *Phys. Chem. Chem. Phys.* **20**, 6591–6597 (2018).

25. D. Hu, D. Topping, G. McFiggans, Measured particle water uptake enhanced by co-condensing vapours. *Atmospheric Chem. Phys.* **18**, 14925–14937 (2018).

26. L. N. Hawkins, L. M. Russell, Oxidation of ketone groups in transported biomass burning aerosol from the 2008 Northern California Lightning Series fires. *Atmos. Environ.* **44**, 4142–4154 (2010).

27. P. F. Liu, C. S. Zhao, T. Göbel, E. Hallbauer, A. Nowak, L. Ran, W. Y. Xu, Z. Z. Deng, N. Ma, K. Mildenberger, S. Henning, F. Stratmann, A. Wiedensohler, Hygroscopic properties of aerosol particles at high relative humidity and their diurnal variations in the North China Plain. *Atmospheric Chem. Phys.* **11**, 3479–3494 (2011).

28. J. D. Whitehead, M. Irwin, J. D. Allan, N. Good, G. McFiggans, A meta-analysis of particle water uptake reconciliation studies. *Atmospheric Chem. Phys.* **14**, 11833–11841 (2014).

29. K. Kawana, T. Nakayama, M. Mochida, Hygroscopicity and CCN activity of atmospheric aerosol particles and their relation to organics: Characteristics of urban aerosols in Nagoya, Japan. *J. Geophys. Res. Atmospheres* **121**, 4100–4121 (2016).

30. J. H. Kim, S. S. Yum, S. Shim, S.-C. Yoon, J. G. Hudson, J. Park, S.-J. Lee, On aerosol hygroscopicity, cloud condensation nuclei (CCN) spectra and critical supersaturation measured at two remote islands of Korea between 2006 and 2009. *Atmospheric Chem. Phys.* **11**, 12627–12645 (2011).

31. K. M. Cerully, T. Raatikainen, S. Lance, D. Tkacik, P. Tiitta, T. Petäjä, M. Ehn, M. Kulmala, D. R. Worsnop, A. Laaksonen, J. N. Smith, A. Nenes, Aerosol hygroscopicity and CCN activation kinetics in a boreal forest environment during the 2007 EUCAARI campaign. *Atmospheric Chem. Phys.* **11**, 12369–12386 (2011).

32. G. C. Roberts, D. A. Day, L. M. Russell, E. J. Dunlea, J. L. Jimenez, J. M. Tomlinson, D. R. Collins, Y. Shinozuka, A. D. Clarke, Characterization of particle cloud droplet activity and composition in the free troposphere and the boundary layer during INTEX-B. *Atmospheric Chem. Phys.* **10**, 6627–6644 (2010).

33. A. Müller, Y. Miyazaki, S. G. Aggarwal, Y. Kitamori, S. K. R. Boreddy, K. Kawamura, Effects of chemical composition and mixing state on size-resolved hygroscopicity and cloud condensation nuclei activity of submicron aerosols at a suburban site in northern Japan in summer. *J. Geophys. Res. Atmospheres* **122**, 9301–9318 (2017).

34. D. Bates, A. Noack, S. Kornblith, M. Bouchet-Valat, M. K. Borregaard, A. Arslan, J. M. White, D. Kleinschmidt, P. Alday, G. Lynch, I. Dunning, P. K. Mogensen, S. Lendle, D. Aluthge, M. Dutta, pdeffebach, P. José Bayoán Santiago Calderón, A. Patnaik, B. Born, B. Setzler, C. DuBois, J. Quinn, O. Slámečka, P. Bastide, V. B. Shah, P. Anthony Blaom, B. König, JuliaStats/GLM.jl: v1.9.0, Zenodo (2023); https://doi.org/10.5281/zenodo.8345558.

35. S. Romakkaniemi, A. Jaatinen, A. Laaksonen, A. Nenes, T. Raatikainen, Ammonium nitrate evaporation and nitric acid condensation in DMT CCN counters. *Atmospheric Meas. Tech.* **7**, 1377–1384 (2014).

36. X. Tang, D. Price, E. Praske, D. N. Vu, K. Purvis-Roberts, P. J. Silva, D. R. Cocker III, A. Asa-Awuku, Cloud condensation nuclei (CCN) activity of aliphatic amine secondary aerosol. *Atmospheric Chem. Phys.* **14**, 5959–5967 (2014).

37. Y. Kuang, W. Xu, J. Tao, N. Ma, C. Zhao, M. Shao, A review on laboratory studies and field measurements of atmospheric organic aerosol hygroscopicity and its parameterization based on oxidation levels. *Curr. Pollut. Rep.* **6**, 410–424 (2020).

38. A. T. Lambe, T. B. Onasch, P. Massoli, D. R. Croasdale, J. P. Wright, A. T. Ahern, L. R. Williams, D. R. Worsnop, W. H. Brune, P. Davidovits, Laboratory studies of the chemical composition and cloud condensation nuclei (CCN) activity of secondary organic aerosol (SOA) and oxidized primary organic aerosol (OPOA). *Atmos. Chem. Phys.* **11**, 8913–8928 (2011).

39. M. D. Petters, A. J. Prenni, S. M. Kreidenweis, P. J. DeMott, A. Matsunaga, Y. B. Lim, P. J. Ziemann, Chemical aging and the hydrophobic-to-hydrophilic conversion of carbonaceous aerosol. *Geophys. Res. Lett.* **33**, doi.org/10.1029/2006GL027249 (2006).

40. I. J. George, J. P. D. Abbatt, Chemical evolution of secondary organic aerosol from OH-initiated heterogeneous oxidation. *Atmos. Chem. Phys.* **10**, 5551–5563 (2010).

41. J. H. Slade, R. Thalman, J. Wang, D. A. Knopf, Chemical aging of single and multicomponent biomass burning aerosol surrogate particles by OH: Implications for cloud condensation nucleus activity. *Atmos. Chem. Phys.* **15**, 10183–10201 (2015).

42. K. E. Broekhuizen, T. Thornberry, P. P. Kumar, J. P. D. Abbatt, Formation of cloud condensation nuclei by oxidative processing: Unsaturated fatty acids. *J. Geophys. Res. Atmospheres* **109**, doi.org/10.1029/2004JD005298 (2004).

43. M. D. Petters, S. M. Kreidenweis, P. J. Ziemann, Prediction of cloud condensation nuclei activity for organic compounds using functional group contribution methods. *Geosci Model Dev* **9**, 111–124 (2016).

44. S. M. Kreidenweis, M. D. Petters, P. Y. Chuang, "Cloud particle precursors" in *Clouds in the Perturbed Climate System: Their Relationship to Energy Balance, Atmospheric Dynamics, and Precipitation*, J. Heintzenberg, R. J. Charlson, Eds. (The MIT Press, 2009;), p. 0, 10.7551/mitpress/9780262012874.003.0013.

45. A. Laaksonen, P. Korhonen, M. Kulmala, R. J. Charlson, Modification of the Köhler equation to include soluble trace gases and slightly soluble substances. *J. Atmos. Sci.* **55**, 853–862 (1998).

46. F. Samie, J. Tidblad, V. Kucera, C. Leygraf, Atmospheric corrosion effects of HNO—Method development and results on laboratory-exposed copper. *Atmos. Environ.* **39**, 7362–7373 (2005).

47. D. L. Fox, L. Stockburger, W. Weathers, C. W. Spicer, G. I. Mackay, H. I. Schiff, D. J. Eatough, F. Mortensen, L. D. Hansen, P. B. Shepson, T. E. Kleindienst, E. O. Edney, Intercomparison of nitric acid diffusion denuder methods with tunable diode laser absorption spectroscopy. *Atmospheric Environ.* **22**, 575–585 (1988).

48. W. P. Hastings, C. A. Koehler, E. L. Bailey, D. O. De Haan, Secondary organic aerosol formation by glyoxal hydration and oligomer formation: Humidity effects and equilibrium shifts during analysis. *Environ. Sci. Technol.* **39**, 8728–8735 (2005).

49. E. Grosjean, D. Grosjean, M. P. Fraser, G. R. Cass, Air quality model evaluation data for organics. 2. C_1−C_{14} Carbonyls in Los Angeles Air. *Environ. Sci. Technol.* **30**, 2687–2703 (1996).

50. C. B. Richardson, R. L. Hightower, Evaporation of ammonium nitrate particles. *Atmos. Environ.* **21**, 971–975 (1987).

51. S. Han, A. S. Williams, L. M. Russell, V. Z. Berta, J. L. Dedrick, C. Pelayo, N. Maneenoi, A. Osawa, I. Silber, D. Zhang, M. A. Zawadowicz, A. J. I. Sedlacek, Nearby sites show similar upwind sources and differing semivolatile concentrations in coastal aerosol particles. *ACS EST Air* **12**, 2824–2837 (2025).

52. A. Tandon, N. E. Rothfuss, M. D. Petters, The effect of hydrophobic glassy organic material on the cloud condensation nuclei activity of particles with different morphologies. *Atmos. Chem. Phys.* **19**, 3325–3339 (2019).

53. Q. T. Nguyen, K. H. Kjær, K. I. Kling, T. Boesen, M. Bilde, Impact of fatty acid coating on the CCN activity of sea salt particles. *Tellus B, Chem. Phys. Meteor.* **69**, 1304064 (2022).

54. B. R. Bzdek, R. M. Power, S. H. Simpson, J. P. Reid, C. P. Royall, Precise, contactless measurements of the surface tension of picolitre aerosol droplets. *Chem. Sci.* **7**, 274–285 (2016).

55. C. R. Ruehl, J. F. Davies, K. R. Wilson, An interfacial mechanism for cloud droplet formation on organic aerosols. *Science* **351**, 1447–1450 (2016).

56. D. J. Donaldson, K. T. Valsaraj, Adsorption and reaction of trace gas-phase organic compounds on atmospheric water film surfaces: A critical review. *Environ. Sci. Technol.* **44**, 865–873 (2010).

57. H. Hao, I. Leven, T. Head-Gordon, Can electric fields drive chemistry for an aqueous microdroplet? *Nat. Commun.* **13**, 280 (2022).

58. P. Pourhakkak, M. Taghizadeh, A. Taghizadeh, M. Ghaedi, "Chapter 2 - Adsorbent" in *Interface Science and Technology*, M. Ghaedi, Ed. (Elsevier, 2021; https://sciencedirect.com/science/article/pii/B9780128188057000096) vol. 33 of *Adsorption: Fundamental Processes and Applications*, pp. 71–210.

59. S. Romakkaniemi, H. Kokkola, A. Laaksonen, Soluble trace gas effect on cloud condensation nuclei activation: Influence of initial equilibration on cloud model results. *J. Geophys. Res. Atmospheres* **110**, doi.org/10.1029/2004JD005364 (2005).

60. H. Kokkola, S. Romakkaniemi, A. Laaksonen, Köhler theory for a polydisperse droplet population in the presence of a soluble trace gas, and an application to stratospheric STS droplet growth. *Atmos. Chem. Phys.* **3**, 2139–2146 (2003).

61. H. Kokkola, S. Romakkaniemi, A. Laaksonen, M. Kulmala, A cloud microphysics model including trace gas condensation and sulfate chemistry. *Boreal Environ. Res.* **8**, 413–424 (2003).

62. R. Sorjamaa, B. Svenningsson, T. Raatikainen, S. Henning, M. Bilde, A. Laaksonen, The role of surfactants in Köhler theory reconsidered. *Atmos. Chem. Phys.* **4**, 2107–2117 (2004).

63. H. Wex, F. Stratmann, D. Topping, G. McFiggans, The Kelvin versus the Raoult term in the Köhler equation. *J. Atmos. Sci.* **65**, 4004–4016 (2008).

64. U. Dusek, G. P. Frank, A. Massling, K. Zeromskiene, Y. Iinuma, O. Schmid, G. Helas, T. Hennig, A. Wiedensohler, M. O. Andreae, Water uptake by biomass burning aerosol at sub- and supersaturated conditions: Closure studies and implications for the role of organics. *Atmos. Chem. Phys.* **11**, 9519–9532 (2011).

65. M. Irwin, N. Good, J. Crosier, T. W. Choularton, G. McFiggans, Reconciliation of measurements of hygroscopic growth and critical supersaturation of aerosol particles in central Germany. *Atmos. Chem. Phys.* **10**, 11737–11752 (2010).

66. S. D. Forestieri, S. M. Staudt, T. M. Kuborn, K. Faber, C. R. Ruehl, T. H. Bertram, C. D. Cappa, Establishing the impact of model surfactants on cloud condensation nuclei activity of sea spray aerosol mimics. *Atmos. Chem. Phys.* **18**, 10985–11005 (2018).

67. N. Tankovsky, N. Zografov, A. Andreeva, Gas-adsorption dynamics at the water–air interface, revealed by resonant droplet tensiometry. *Chem. Eng. Sci.* **144**, 283–287 (2016).

68. R. G. Bruant, “Adsorption of volatile hydrophobic organic compounds at the vapor /water interface,” thesis, The University of Arizona, Tucson, Arizona (2000).

69. D. J. Donaldson, Adsorption of Atmospheric Gases at the air−water interface. I. NH3. *J. Phys. Chem. A* **103**, 62–70 (1999).

70. D. J. Donaldson, D. Anderson, Adsorption of atmospheric gases at the air−water interface. 2. C_1−C_4 Alcohols, acids, and acetone. *J. Phys. Chem. A* **103**, 871–876 (1999).

71. C. E. Kolb, R. A. Cox, J. P. D. Abbatt, M. Ammann, E. J. Davis, D. J. Donaldson, B. C. Garrett, C. George, P. T. Griffiths, D. R. Hanson, M. Kulmala, G. McFiggans, U. Pöschl, I. Riipinen, M. J. Rossi, Y. Rudich, P. E. Wagner, P. M. Winkler, D. R. Worsnop, C. D. O'Dowd, An overview of current issues in the uptake of atmospheric trace gases by aerosols and clouds. *Atmos. Chem. Phys.* **10**, 10561–10605 (2010).

72. J. Ovadnevaite, A. Zuend, A. Laaksonen, K. J. Sanchez, G. Roberts, D. Ceburnis, S. Decesari, M. Rinaldi, N. Hodas, M. C. Facchini, J. H. Seinfeld, C. O'Dowd, Surface tension prevails over solute effect in organic-influenced cloud droplet activation. *Nature* **546**, 637–641 (2017).

73. P. Liu, M. Song, T. Zhao, S. S. Gunthe, S. Ham, Y. He, Y. M. Qin, Z. Gong, J. C. Amorim, A. K. Bertram, S. T. Martin, Resolving the mechanisms of hygroscopic growth and cloud condensation nuclei activity for organic particulate matter. *Nat. Commun.* **9**, 4076 (2018).

74. A. Pajunoja, A. T. Lambe, J. Hakala, N. Rastak, M. J. Cummings, J. F. Brogan, L. Hao, M. Paramonov, J. Hong, N. L. Prisle, J. Malila, S. Romakkaniemi, K. E. J. Lehtinen, A. Laaksonen, M. Kulmala, P. Massoli, T. B. Onasch, N. M. Donahue, I. Riipinen, P. Davidovits, D. R. Worsnop, T. Petäjä, A. Virtanen, Adsorptive uptake of water by semisolid secondary organic aerosols. *Geophys. Res. Lett.* **42**, 3063–3068 (2015).

75. M. L. Shulman, M. C. Jacobson, R. J. Carlson, R. E. Synovec, T. E. Young, Dissolution behavior and surface tension effects of organic compounds in nucleating cloud droplets. *Geophys. Res. Lett.* **23**, 277–280 (1996).

76. R. Zhang, A. F. Khalizov, J. Pagels, D. Zhang, H. Xue, P. H. McMurry, Variability in morphology, hygroscopicity, and optical properties of soot aerosols during atmospheric processing. *Proc. Natl. Acad. Sci. U.S.A.* **105**, 10291–10296 (2008).

77. F. Zhang, Y. Wang, J. Peng, J. Ren, D. Collins, R. Zhang, Y. Sun, X. Yang, Z. Li, Uncertainty in predicting CCN activity of aged and primary aerosols. *J. Geophys. Res. Atmospheres* **122**, 11,723–11,736 (2017).

78. Fofie. Emmanuel A., G. Karavalakis, A. Asa-Awuku, R. "Cloud forming potential of aerosol from light-duty gasoline direct injection vehicles" (Univ. of California, 2017); https://rosap.ntl.bts.gov/view/dot/34571.

79. X. Liu, J. Wang, How important is organic aerosol hygroscopicity to aerosol indirect forcing? *Environ. Res. Lett.* **5**, 044010 (2010).

80. M. Petters, E. Ravichandran, A. S. Williams, S. Han, C. Pelayo, J. L. Dedrick, L. M. Russell, (2023). Cloud Condensation Nuclei and Printed Optical Particle Spectrometer Measurements at Mt. Soledad during EPCAPE 2023–24. In Aerosol Microphysics and Chemical Measurements at Mt. Soledad and Scripps Pier during the Eastern Pacific Cloud Aerosol Precipitation Experiment (EPCAPE) from February 2023 to February 2024. UC San Diego Library Digital Collections. https://doi.org/10.6075/J0KH0NHG.

81. S. S. Petters, M. D. Petters, Surfactant effect on cloud condensation nuclei for two-component internally mixed aerosols. *J. Geophys. Res. Atmos.* **121**, 1878–1895 (2016).

82. A. C. Martin, G. C. Cornwell, S. A. Atwood, K. A. Moore, N. E. Rothfuss, H. Taylor, P. J. DeMott, S. M. Kreidenweis, M. D. Petters, K. A. Prather, Transport of pollution to a remote coastal site during gap flow from California's interior: Impacts on aerosol composition, clouds, and radiative balance. *Atmos. Chem. Phys.* **17**, 1491–1509 (2017).

83. T. S. Bates, D. J. Coffman, D. S. Covert, P. K. Quinn, Regional marine boundary layer aerosol size distributions in the Indian, Atlantic, and Pacific Oceans: A comparison of INDOEX measurements with ACE-1, ACE-2, and Aerosols99. *J. Geophys. Res. Atmos.* **107**, INX2 25-1–INX2 25-15 (2002).

84. P. Mikuška, Z. Večeřa, A. Bartošíková, W. Maenhaut, Annular diffusion denuder for simultaneous removal of gaseous organic compounds and air oxidants during sampling of carbonaceous aerosols. *Anal. Chim. Acta* **714**, 68–75 (2012).

85. M. D. Petters, Revisiting matrix-based inversion of scanning mobility particle sizer (SMPS) and humidified tandem differential mobility analyzer (HTDMA) data. *Atmos. Meas. Tech.* **14**, 7909–7928 (2021).

86. M. D. Petters, A language to simplify computation of differential mobility analyzer response functions. *Aerosol Sci. Technol.* **52**, 1437–1451 (2018).

87. S. I. Christensen, M. D. Petters, The role of temperature in cloud droplet activation. *J. Phys. Chem. A* **116**, 9706–9717 (2012).

88. Atmospheric Radiation Measurement (ARM) user facility. 2021. Humidified Tandem Differential Mobility Analyzer (AOSHTDMA). 2021-09-13 to 2021-12-02, ARM Mobile Facility (HOU) Houston, TX; AMF1 (main site for TRACER) (M1). Compiled by J. Uin, E. Cromwell, C. Hayes and C. Salwen. ARM Data Center. Data set accessed 2024-01-22 at http://dx.doi.org/10.5439/1776643.

89. M. D. Petters, A software package to simplify Tikhonov regularization with examples for matrix-based multi-charge inversion of SMPS and HTDMA data. (2021).

90. K. L. Hayden, S.-M. Li, J. Liggio, M. J. Wheeler, J. J. B. Wentzell, A. Leithead, P. Brickell, R. L. Mittermeier, Z. Oldham, C. M. Mihele, R. M. Staebler, S. G. Moussa, A. Darlington, M. Wolde, D. Thompson, J. Chen, D. Griffin, E. Eckert, J. C. Ditto, M. He, D. R. Gentner, Reconciling the total carbon budget for boreal forest wildfire emissions using airborne observations. *Atmos. Chem. Phys.* **22**, 12493–12523 (2022).

91. C. Young, S. Joudan, Y. Tao, J. Wentzell, J. Liggio, High time resolution ambient observations of gas-phase perfluoroalkyl carboxylic acids: Implications for atmospheric sources. ChemRxiv [Preprint] (2024). https://doi.org/10.26434/chemrxiv-2024-cd158.

92. Y. Ji, L. G. Huey, D. J. Tanner, Y. R. Lee, P. R. Veres, J. A. Neuman, Y. Wang, X. Wang, A vacuum ultraviolet ion source (VUV-IS) for iodide–chemical ionization mass spectrometry: A substitute for radioactive ion sources. *Atmos. Meas. Tech.* **13**, 3683–3696 (2020).

93. M. Breitenlechner, G. A. Novak, J. A. Neuman, A. W. Rollins, P. R. Veres, A versatile vacuum ultraviolet ion source for reduced pressure bipolar chemical ionization mass spectrometry. *Atmos. Meas. Tech.* **15**, 1159–1169 (2022).

94. M. Riva, V. Pospisilova, C. Frege, S. Perrier, P. Bansal, S. Jorga, P. Sturm, J. A. Thornton, U. Rohner, F. Lopez-Hilfiker, Evaluation of a reduced-pressure chemical ion reactor utilizing adduct ionization for the detection of gaseous organic and inorganic species. *Atmos. Meas. Tech.* **17**, 5887–5901 (2024).

95. I. Silber, J. M. Comstock, M. R. Kieburtz, L. M. Russell, ARMTRAJ: A set of multipurpose trajectory datasets augmenting the Atmospheric Radiation Measurement (ARM) user facility measurements. *Earth Syst. Sci. Data* **17**, 29–42 (2025).

96. H. Hersbach, B. Bell, P. Berrisford, S. Hirahara, A. Horányi, J. Muñoz-Sabater, J. Nicolas, C. Peubey, R. Radu, D. Schepers, A. Simmons, C. Soci, S. Abdalla, X. Abellan, G. Balsamo, P. Bechtold, G. Biavati, J. Bidlot, M. Bonavita, G. De Chiara, P. Dahlgren, D. Dee, M. Diamantakis, R. Dragani, J. Flemming, R. Forbes, M. Fuentes, A. Geer, L. Haimberger, S. Healy, R. J. Hogan, E. Hólm, M. Janisková, S. Keeley, P. Laloyaux, P. Lopez, C. Lupu, G. Radnoti, P. de Rosnay, I. Rozum, F. Vamborg, S. Villaume, J.-N. Thépaut, The ERA5 global reanalysis. *Q. J. R. Meteorol. Soc.* **146**, 1999–2049 (2020).

97. M. T. Markou, P. Kassomenos, Cluster analysis of five years of back trajectories arriving in Athens, Greece. *Atmos. Res.* **98**, 438–457 (2010).

98. V. Berta, S. Han, A. Osawa, J. Dedrick, N. Maneenoi, L. M. Russell, Aerosol quantification products at Mt. Soledad and Scripps Pier during EPCAPE 2023–24, in *Aerosol Microphysics and Chemical Measurements at Mt. Soledad and Scripps Pier during the Eastern Pacific Cloud Aerosol Precipitation Experiment (EPCAPE)* from February 2023 to February 2024, UC San Diego Library Digital Collections (2025); https://doi.org/10.6075/J0WM1DSC.

99. A. S. Williams, V. Berta, S. Han, L. M. Russell (2023). Online chemical composition of submicron aerosol particles and cloud residuals at Mt. Soledad during EPCAPE 2023–24, in *Aerosol Microphysics and Chemical Measurements at Mt. Soledad and Scripps Pier during the Eastern Pacific Cloud Aerosol Precipitation Experiment (EPCAPE)* from February 2023 to February 2024. UC San Diego Library Digital Collections. https://doi.org/10.6075/J0ZS2WQ0.

100. J. Liggio, J. Wentzell, M. Wheeler (2025). Online gas-phase organic and inorganic chemical composition at Mt. Soledad during EPCAPE 2023–24, in *Aerosol Microphysics and*

Chemical Measurements at Mt. Soledad and Scripps Pier during the Eastern Pacific Cloud Aerosol Precipitation Experiment (EPCAPE) from February 2023 to February 2024. UC San Diego Library Digital Collections. https://doi.org/10.6075/J0RV0P3H.

101. C. Yaws, "Chapter 10. Diffusion coefficient in air – organic compounds" in *Transport Properties of Chemicals and Hydrocarbons* (2009), pp. 502–593.

102. R. B. Bird, Transport phenomena. *Appl. Mech. Rev.* **55**, R1–R4 (2002).

103. A. L. Lydersen, *Estimation of Critical Properties of Organic Compounds by the Method of Group Contibutions* (Univ. of Wisconsin, Madison, 1955) Report.

104. S. Kim, J. Chen, T. Cheng, A. Gindulyte, J. He, S. He, Q. Li, B. A. Shoemaker, P. A. Thiessen, B. Yu, L. Zaslavsky, J. Zhang, E. E. Bolton, PubChem 2023 update. *Nucleic Acids Res.* **51**, D1373–D1380 (2023).

105. M. D. Petters, A. J. Prenni, S. M. Kreidenweis, P. J. DeMott, On measuring the critical diameter of cloud condensation nuclei using mobility selected aerosol. *Aerosol Sci. Technol.* **41**, 907–913 (2007).

106. A. S. Wexler, S. L. Clegg, Atmospheric aerosol models for systems including the ions H+, NH4+, Na+, SO_{42}−, NO_3−, Cl−, Br−, and H_2O. *J. Geophys. Res. Atmospheres* **107**, ACH 14-1–ACH 14-14 (2002).

107. E. J. T. Levin, A. J. Prenni, M. D. Petters, S. M. Kreidenweis, R. C. Sullivan, S. A. Atwood, J. Ortega, P. J. DeMott, J. N. Smith, An annual cycle of size-resolved aerosol hygroscopicity at a forested site in Colorado. *J. Geophys. Res. Atmospheres* **117**, doi. org/10.1029/2011JD016854 (2012).

108. M. Bilde, B. Svenningsson, J. Mønster, T. Rosenørn, Even−odd alternation of evaporation rates and vapor pressures of C3−C9 dicarboxylic acid aerosols. *Environ. Sci. Technol.* **37**, 1371–1378 (2003).

109. R. Sander, Compilation of Henry's law constants (version 4.0) for water as solvent. *Atmos. Chem. Phys.* **15**, 4399–4981 (2015).

110. R. Sander, Modeling atmospheric chemistry: Interactions between gas-phase species and liquid cloud/aerosol particles. *Surv. Geophys.* **20**, 1–31 (1999).

111. S. Kasparoglu, L. Cai, N. Meskhidze, M. D. Petters, Evolution of refractory black carbon mixing state in an urban environment. *Atmos. Environ.* **333**, 120651 (2024).
